# Genotype-Phenotype Taxonomy of Hypertrophic Cardiomyopathy

**DOI:** 10.1161/CIRCGEN.123.004200

**Published:** 2023-11-28

**Authors:** Lara Curran, Antonio de Marvao, Paolo Inglese, Kathryn A. McGurk, Pierre-Raphaël Schiratti, Adam Clement, Sean L. Zheng, Surui Li, Chee Jian Pua, Mit Shah, Mina Jafari, Pantazis Theotokis, Rachel J. Buchan, Sean J. Jurgens, Claire E. Raphael, Arun John Baksi, Antonis Pantazis, Brian P. Halliday, Dudley J. Pennell, Wenjia Bai, Calvin W.L. Chin, Rafik Tadros, Connie R. Bezzina, Hugh Watkins, Stuart A. Cook, Sanjay K. Prasad, James S. Ware, Declan P. O’Regan

**Affiliations:** 1 National Heart and Lung Institute (L.C., K.A.M., S.L.Z., P.T., R.J.B., C.E.R., A.J.B., A.P., B.P.H., D.J.P., S.K.P., J.S.W.); 2 Biomedical Image Analysis Group, Department of Computing (S.L., W.B.); 3 Department of Brain Sciences, Imperial College London, London, United Kingdom (W.B.).; 4 Royal Brompton and Harefield Hospitals, Guy’s and St. Thomas’ NHS Foundation Trust (L.C., R.J.B., C.E.R., A.J.B., A.P., B.P.H., D.J.P., S.K.P., J.S.W.).; 5 Medical Research Council Laboratory of Medical Sciences, Imperial College London, United Kingdom (A.d.M., P.I., K.A.M., P.-R.S., A.C., S.L.Z., S.L., M.S., M.J., P.T., R.J.B., S.A.C., J.S.W., D.P.O.).; 6 Department of Women and Children’s Health (A.d.M.).; 7 British Heart Foundation Centre of Research Excellence, School of Cardiovascular & Metabolic Medicine and Sciences, King’s College London, United Kingdom (A.d.M.).; 8 National Heart Research Institute Singapore, Singapore, PRC (C.J.P., C.W.L.C., S.A.C.).; 9 Department of Cardiology, National Heart Center Singapore, Singapore, PRC (C.W.L.C.).; 10 Cardiovascular Sciences ACP, Duke NUS Medical School, Singapore (C.W.L.C.).; 11 Mayo Clinic Rochester, MN (C.E.R.).; 12 Department of Experimental Cardiology, Heart Center, Amsterdam Cardiovascular Sciences, Amsterdam University Medical Centers, University of Amsterdam, the Netherlands (S.J.J., C.R.B.).; 13 Cardiovascular Disease Initiative, Broad Institute of MIT and Harvard, Cambridge, MA (S.J.J.).; 14 Cardiovascular Genetics Centre, Montreal Heart Institute (R.T.).; 15 Faculty of Medicine, Université de Montréal, QC, Canada (R.T.).; 16 Radcliffe Department of Medicine, University of Oxford, United Kingdom (H.W.).

**Keywords:** genotype, hypertension, hypertrophy, magnetic resonance imaging, phenotype

## Abstract

**BACKGROUND:**

Hypertrophic cardiomyopathy (HCM) is an important cause of sudden cardiac death associated with heterogeneous phenotypes, but there is no systematic framework for classifying morphology or assessing associated risks. Here, we quantitatively survey genotype-phenotype associations in HCM to derive a data-driven taxonomy of disease expression.

**METHODS:**

We enrolled 436 patients with HCM (median age, 60 years; 28.8% women) with clinical, genetic, and imaging data. An independent cohort of 60 patients with HCM from Singapore (median age, 59 years; 11% women) and a reference population from the UK Biobank (n=16 691; mean age, 55 years; 52.5% women) were also recruited. We used machine learning to analyze the 3-dimensional structure of the left ventricle from cardiac magnetic resonance imaging and build a tree-based classification of HCM phenotypes. Genotype and mortality risk distributions were projected on the tree.

**RESULTS:**

Carriers of pathogenic or likely pathogenic variants for HCM had lower left ventricular mass, but greater basal septal hypertrophy, with reduced life span (mean follow-up, 9.9 years) compared with genotype negative individuals (hazard ratio, 2.66 [95% CI, 1.42–4.96]; *P*<0.002). Four main phenotypic branches were identified using unsupervised learning of 3-dimensional shape: (1) nonsarcomeric hypertrophy with coexisting hypertension; (2) diffuse and basal asymmetrical hypertrophy associated with outflow tract obstruction; (3) isolated basal hypertrophy; and (4) milder nonobstructive hypertrophy enriched for familial sarcomeric HCM (odds ratio for pathogenic or likely pathogenic variants, 2.18 [95% CI, 1.93–2.28]; *P*=0.0001). Polygenic risk for HCM was also associated with different patterns and degrees of disease expression. The model was generalizable to an independent cohort (trustworthiness, M_1_: 0.86–0.88).

**CONCLUSIONS:**

We report a data-driven taxonomy of HCM for identifying groups of patients with similar morphology while preserving a continuum of disease severity, genetic risk, and outcomes. This approach will be of value in understanding the causes and consequences of disease diversity.

Hypertrophic cardiomyopathy (HCM) is an inherited cardiac condition (prevalence ≈1 in 500) related to increased risk of sudden death and adverse cardiac events, including in early life and middle age, which is associated with genetic and phenotypic heterogeneity.^1^ Although traditionally considered a Mendelian disease, polygenic variation is now recognized as contributing to phenotypic variability in carriers of HCM-causing rare sarcomeric variants.^2,3^ Sex and environmental risk factors also interact with disease-associated variants to modify susceptibility.^4^ HCM-associated rare variants are not infrequently observed in the general population, but the most prevalent variants cause an attenuated phenotype and lower risk of adverse events outside the context of familial disease.^5^ In the more common nonsarcomeric HCM, there is substantial polygenic inheritance and modifiable risk factors have important roles in disease expressivity.^3^ HCM is also not a static condition and adverse remodeling and fibrosis can evolve over time.^6^ Such dynamic endophenotypic diversity presents challenges for understanding drivers of heterogeneity, identifying patients enriched for pathogenic variants, and for developing personalized clinical profiles to guide intervention.

Current approaches to improve patient stratification in HCM, using sarcomere variant status and morphological traits, have described potential functional and anatomic groupings with differing outcomes.^7,8^ However, such strategies do not align with the molecular understanding of HCM as a continuum of phenotypic expression influenced by genetic and environmental modifiers.^9^ While optimal care requires cardiac imaging to confirm a diagnosis of HCM and characterize individual pathophysiology,^10^ there is limited understanding of phenotypic diversity and its relevance to genotype status and clinical management. Our aim was to learn a classification of HCM traits that represents its phenotypic and genetic diversity. Here, we used computer vision to create patient-specific 3-dimensional (3D) models of the left ventricle (LV) from routine cardiac magnetic resonance (CMR) imaging (Figure 1A). First, we used statistical shape modeling to visualize genotype-phenotype associations in patients with HCM compared with a control population from the UK Biobank (UKB). Second, we used unsupervised machine learning to discover natural groupings of HCM phenotypes and represented them in a tree-based taxonomy where distinct morphologies diverge from each other while preserving continuous stratification within each branch. We show how both common and rare variants explain the diversity and severity of phenotypic expression in the tree structure, as well as the relationship to outcomes. We also validated the findings in a small external cohort of patients with HCM. We propose this as a data-driven framework for visualizing individual patient profiles across a spectrum of sarcomeric and nonsarcomeric HCM.

**Figure 1.**
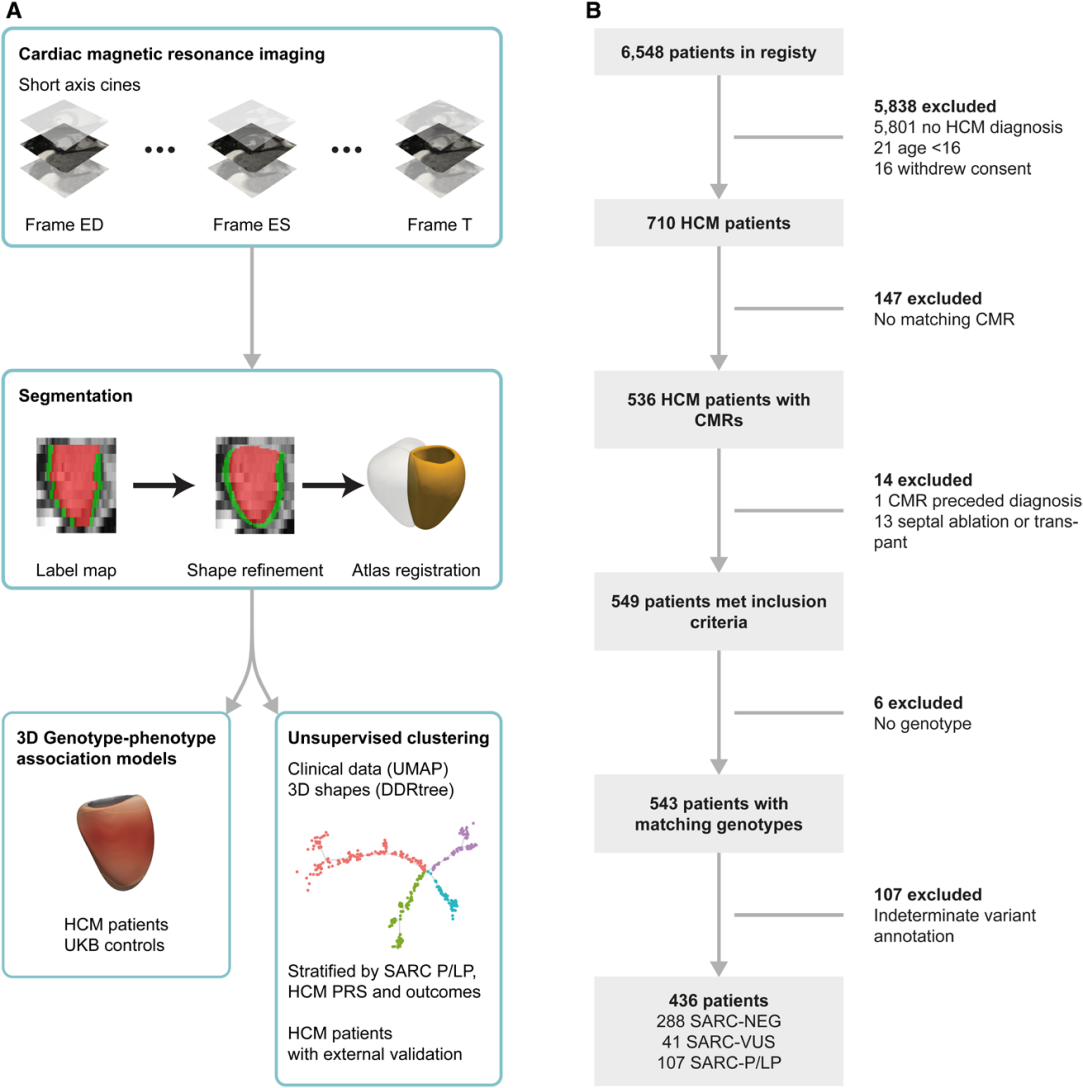
**Study flowchart. A**, Details of the analysis pipeline using segmentations of cardiac magnetic resonance cine imaging to build a 3-dimensional (3D) model of phenotypic variation in UK Biobank and hypertrophic cardiomyopathy (HCM) participants. Association models and clustering analysis is then performed on the data. **B**, Details of patients with HCM recruited to the study and reasons for exclusion. CMR indicates cardiac magnetic resonance; DDRtree, discriminative dimensionality reduction via learning a tree; ED, end diastole; ES, end systole; SARC-NEG, genotype negative; SARC-P/LP, pathogenic/likely pathogenic sarcomeric variants; SARC-VUS, variants of uncertain significance; T, last cardiac phase; UMAP, uniform manifold approximation and projection; UKB, UK Biobank; and PRS, polygenic risk score.

## METHODS

Full methods are available in the Supplemental Material. The code used in the study is available on GitHub (https://github.com/ImperialCollegeLondon/HCM-taxonomy; doi: 10.5281/zenodo.7639435). All raw and derived UKB data are available to approved researchers at https://biobank.ndph.ox.ac.uk/showcase/. The National Research Ethics Service approved the UKB study (11/NW/0382) and the HCM registry (19/SC/0257). The Singaporean study was approved by the Singhealth Centralised Institutional Review Board (2020/2353) and Singhealth Biobank Research Scientific Advisory Executive Committee (SBRSA 2019/001v1). All participants gave written informed consent.

## RESULTS

### Participants

The HCM cohort consisted of 436 eligible patients of whom 287 (66.0%) were classified as genotype negative (SARC-NEG), 41 (9.4%), as carrying sarcomeric variants of uncertain significance (SARC-VUS), and 107 (24.6%) as carrying pathogenic/likely pathogenic (SARC-P/LP) variants (Figure 1B). Most were European (n=352; 80.1%) and men (n=310; 71.1%). Patient demographics and CMR-derived measurements are stratified by genotype in the Table. The great majority of HCM cases were probands (n=423; 97%) and were unrelated (n=426; 98%). Of the remaining nonproband cases (n=13; 3%), 7 were classified as SARC-P/LP, 5 as SARC-NEG, and 1 as SARC-VUS. Data from these patients were used for genotype-phenotype analysis, phenomapping of clinical variables, and tree-based clustering of HCM morphology. For external validation of the tree-based clustering, we used data from 60 Singaporean patients with HCM (Chinese=52, 86.7%; women, 11.7%; median age, 58.9 years; interquartile range [IQR], 46–66), 28 (46.6%) who were classified as SARC-NEG, 16 (26.7%) as SARC-VUS, and 16 (26.7%) as SARC-P/LP (Table S8). For healthy controls in the 3D genotype-phenotype analysis, we used data from 16 691 UKB participants (Table S7) selected for the absence of HCM, and the absence of any rare variant in a gene associated with HCM or a potential genocopy (European=14 683, 87.9%; women, 52.5%; age, 55±7.5 years).

**Table.**
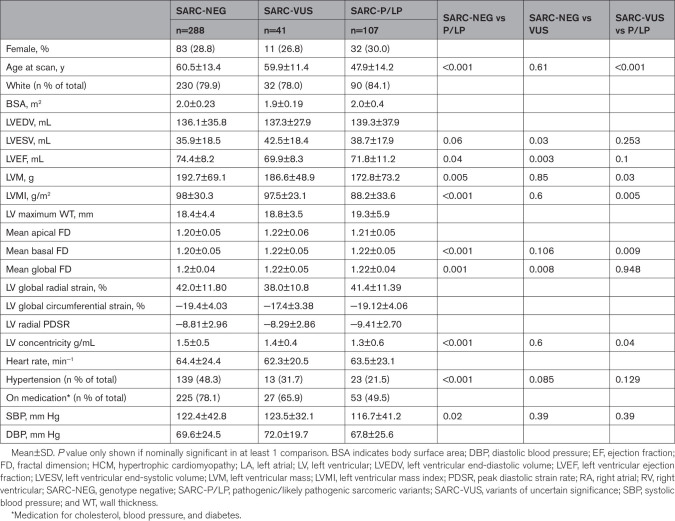
Patient Characteristics and Cardiac Magnetic Resonance Imaging–Derived Measurements by Genotype

### Phenotypes Associated With Variant Status

**Figure 2.**
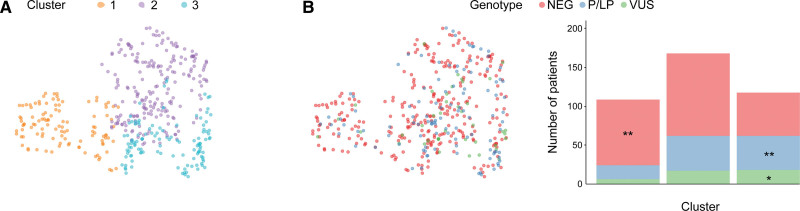
**Unsupervised clustering of patients with hypertrophic cardiomyopathy (HCM) using demographic and anthropometric data, clinical characteristics, and conventional cardiac imaging parameters. A**, Participant clinical features segmented in 3 clusters with a K-means algorithm, optimized with a silhouette score, projected in the 2-dimensional (2D) space of the first 2 uniform manifold approximation and projection (UMAP) components. **B**, Genotype status of participants in the 2D UMAP space with genotype prevalence by cluster. SARC-NEG indicates genotype negative; SARC-P/LP, pathogenic/likely pathogenic sarcomeric variants; and SARC-VUS, variants of uncertain significance. *P≤0.05, **P≤0.01, ***P≤0.001,****P≤0.0001; n=436.

Phenomapping based upon unsupervised clustering of clinical data may help to identify more homogeneous patient phenogroups.^11^ Here, unsupervised analysis of patients’ demographic and anthropometric data, clinical characteristics, and cardiac imaging parameters (Table S11) was performed using uniform manifold approximation and projection and an optimized K-means algorithm. Imaging data included LV mass, volumes, wall thickness, presence of late enhancement, and outflow tract obstruction. Three clusters were identified that were enriched for the following features (Figure 2): (1) females with low BSA, moderate hypertrophy, and hypertension; (2) males with high BSA, more severe hypertrophy, and hypertension; and (3) younger patients with a family history of HCM, no cardiovascular risk factors, and enrichment for SARC-P/LP variants.

Patients with HCM with SARC-P/LP variants had lower LV mass (173±73.2 versus 193±69.1 g; *P*=0.005) and less concentric remodeling (1.3±0.6 versus 1.5±0.5 g/mL; *P*<0.001) than patients with SARC-NEG HCM. Patients with SARC-P/LP also showed increased trabeculation (fractal dimension, 1.22±0.04 versus 1.20±0.04; *P*=0.001) compared with patients with SARC-NEG. Patients with SARC-NEG HCM had a higher prevalence of drug-controlled hypertension than patients with SARC-P/LP variants (48.3% versus 21.5%; *P*<0.001). Differences in end-diastolic and end-systolic volumes were not significant. There were 13 identified nonproband cases.

**Figure 3.**
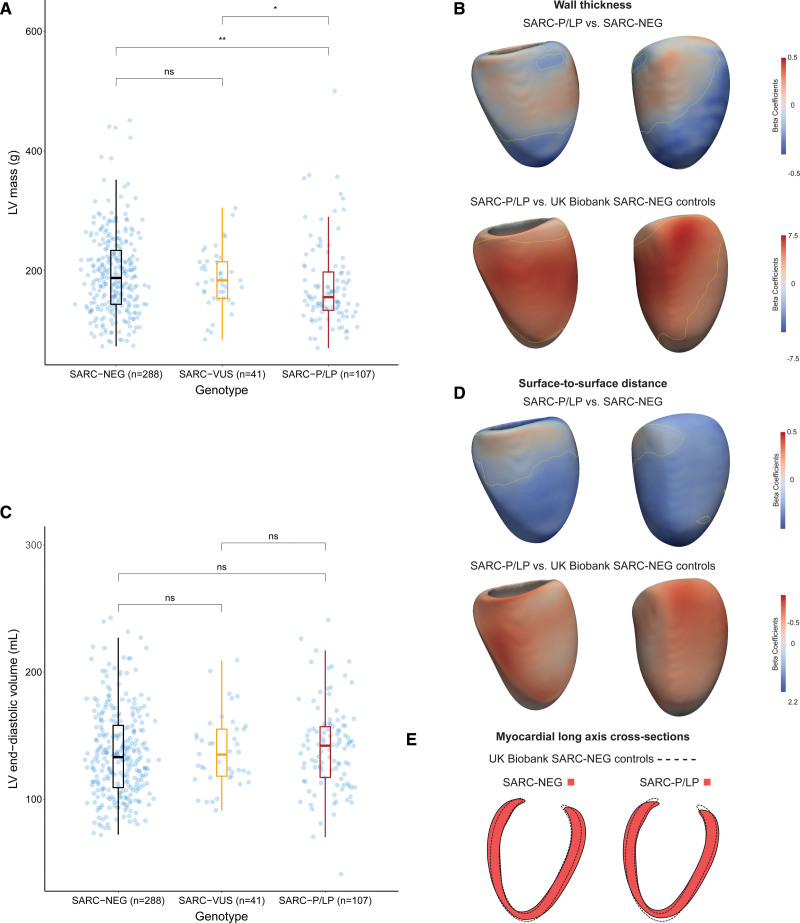
**Genotype-phenotype associations in hypertrophic cardiomyopathy (HCM). A** and **C**, Dot and boxplots of left ventricular (LV) mass and end-diastolic volume in patients with HCM stratified by genotype. SARC-NEG indicates genotype negative; SARC-P/LP, pathogenic/likely pathogenic sarcomeric variants; and SARC-VUS, variants of uncertain significance. **B** and **D**, Three-dimensional modeling of LV geometry with vertex-wise standardized β-coefficients projected on the epicardial surface. These show the extent of association between genotype and wall thickness or surface-to-surface distance (comparing regional shape change) for different comparisons, adjusting for the covariates of age, sex, and race. Yellow contour lines show significant regions (*P*<0.05) after multiple testing correction. LV projections are septal (**left**) and anterior (**right**). **E**, Long-axis cross sections showing the myocardial outline in red for each genotype compared with control participants in UK Biobank (dashed outline). *P≤0.05, **P≤0.01.

We then used 3D analysis of cardiac geometry to compare patterns of remodeling according to variant status. This approach aligns phenotypic data to a common reference space that enables point-by-point linear regression to map the regional effects of variants on shape and wall thickness. This registration allows comparisons both within HCM cohorts and between HCM and genotype-negative control participants in UKB. We found that patients with SARC-P/LP variants had a global increase in wall thickness compared with healthy controls predominantly affecting the basal septum (Figure 3A and 3B). Within the HCM cohort, patients with SARC-P/LP had lower wall thickness across the LV, apart from the basal septum, when compared with those who were SARC-NEG. Although global cavity volumes were similar between genotypes, the 3D models showed smaller ventricular cavity size in SARC-NEG individuals in all but the basal septal segments (Figure 3C through 3E). Taken together, these observations suggest that regional changes in geometry and wall thickness observed in 3D cardiac models may be more sensitive markers of genotype status than traditional measurements of LV mass and volume.

### Taxonomy of HCM Morphology

We used unsupervised machine learning (discriminative dimensionality reduction via learning a tree) to identify clusters of similar LV geometries in HCM. This approach learns a tree-like disease taxonomy where naturally similar morphologies are grouped in the same branch and become progressively more differentiated.^12^ This finds stable clusters from complex morphological traits and preserves a continuous ranking of disease expression. Modeling was performed at both end diastole and end systole to capture structural and functional traits. Significantly associated continuous variables, genotypes, and outcomes are shown for each branch. Internal validation resulted in a good stability of the partitions (Tables S1 and S2).

**Figure 4.**
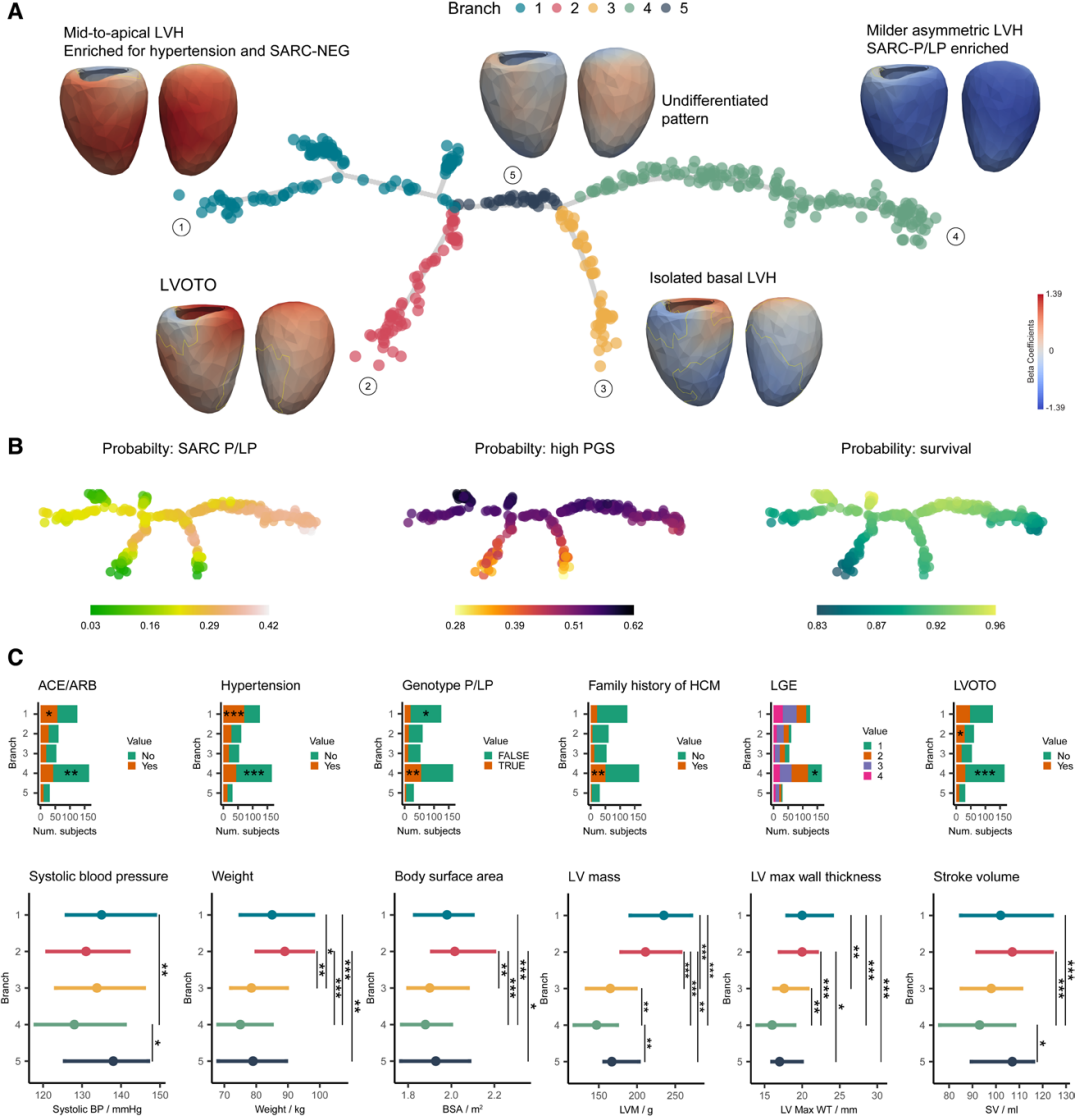
**Phenotypic tree of hypertrophic cardiomyopathy (HCM) morphology. A**, Three-dimensional models of the left ventricle in patients with HCM were reduced to a 2-dimensional tree structure where each point represents 1 individual. The tree maps undifferentiated states in the center to more characteristic morphologies in the distal branches while preserving a continuous stratification. For each branch, we show a wall thickness shape model at end systole where the colors represent β-coefficients for the comparison between branches. Corresponding features at end diastole are shown in Figure S1. Branch 5 showed an undifferentiated mixed phenotype. **B**, Each participant in the tree labeled by probability of pathogenic/likely pathogenic sarcomeric variant (SARC-P/LP) genotype, polygenic score (PGS) for HCM, and predicted survival probability at median age. **C**, Continuous and discrete phenotypic variables found to be significantly associated to at least 1 branch. For late gadolinium enhancement, labels are as follows: 1, none; 2, minimal; 3, moderate, and 4, severe. The significance for the enrichment of discrete variables is reported within the bars. ACE indicates angiotensin-converting enzyme inhibitor; ARB, angiotensin receptor blocker; BP, blood pressure; LGE, late gadolinium enhancement; LV, left ventricular; LVH, left ventricular hypertrophy; LVOTO, left ventricular outflow tract obstruction; SARC-NEG, genotype negative; and SV, stroke volume. Only the significant pairs are reported with the symbols: *P≤0.05, **P≤0.01, ***P≤0.001, ****P≤0.0001; n=436.

The mapping identified 4 main phenotypic branches (Figure 4; Figures S1 and S4). Variation between these was characterized by comparing average LV morphology in the respective branches to all other patients and assessing prevalence of genotype status, as well as other imaging and clinical features. Taken together, enriched characteristics in each branch are summarized as the following: (1) nonsarcomeric mid-to-apical hypertrophy associated with controlled hypertension and fibrosis, (2) diffuse and basal asymmetrical hypertrophy associated with outflow tract obstruction, (3) isolated basal hypertrophy, (4) milder nonobstructive hypertrophy enriched for familial sarcomeric HCM. An additional branch with an undifferentiated pattern of hypertrophy was also identified.

We then explored potential causal processes underlying phenotypic heterogeneity and the relationship to outcomes. Phenotypic variation and associated risks can be visualized as a continuous distribution across the whole taxonomic tree, which we show for genotype status, polygenic score (PGS), and survival (Figure 4B), with end-systolic morphology having the greater overall discrimination (Table S3; Figure S5). The tree structure shows how common and rare variants associated with HCM are differentially enriched in the morphological branches and contribute to continuous phenotypic expression. For instance, there is enrichment of P/LP, decreased survival, and lower LV mass present on the right side of the tree (branch 4). The median odds ratio for carrying P/LP variants in this branch compared with branch 1 is 2.18 ([95% CI, 1.93–2.28], *P*=0.0001). A higher HCM PGS is associated with SARC-NEG status (*P*=0.0012) compared with SARC-P/LP. The taxonomy shows a continuous relationship between the predicted probability of high PGS and individual coordinates on the tree as shown in Figure 4B. Using a logistic regression model with tree coordinates as independent variables, we observed an association between the vertical axis of the tree and PGS (*P*=0.0025) showing lower PGS in more differentiated isolated basal left ventricular hypertrophy and left ventricular outflow tract obstruction-enriched phenotypes.

### External Validation

New patients with imaging can be mapped to coordinates in the tree structure to visualize individual risk and morphological differentiation. We mapped an independent external cohort of patients with HCM with CMR imaging and assessed the similarity of phenotypic patterns. Predictive random forest models for the 2 tree coordinates were tested on the development cohort with 10-fold cross-validation, repeated 3×. Both models had good performances (end diastole: $R_{x}^{2}=0.978$, $R_{y}^{2}=0.952$; end systole: $R_{x}^{2}=0.974$, $R_{y}^{2}=0.897$; for x and y coordinates, respectively). These models were used to predict the tree coordinates of the external HCM cases from their wall thickness values adjusted for sex and age at scan.

Visual inspection of the predicted tree coordinates showed no outliers, with the points falling close to the tree structure (Figure S6). Branch labels were assigned from the nearest neighbor in the development cohort (Table S4). This preserved the faithfulness of the mapping, as confirmed by the estimated trustworthiness^13^ (Supplemental Material; M_1_: end-diastole range, 0.83–0.87; end-systole range, 0.86–0.88). No significant difference was found in the distribution of correlations within 2 cohorts supporting consistency between their projections on the tree (Figure S8).

### Clinical Outcomes

**Figure 5.**
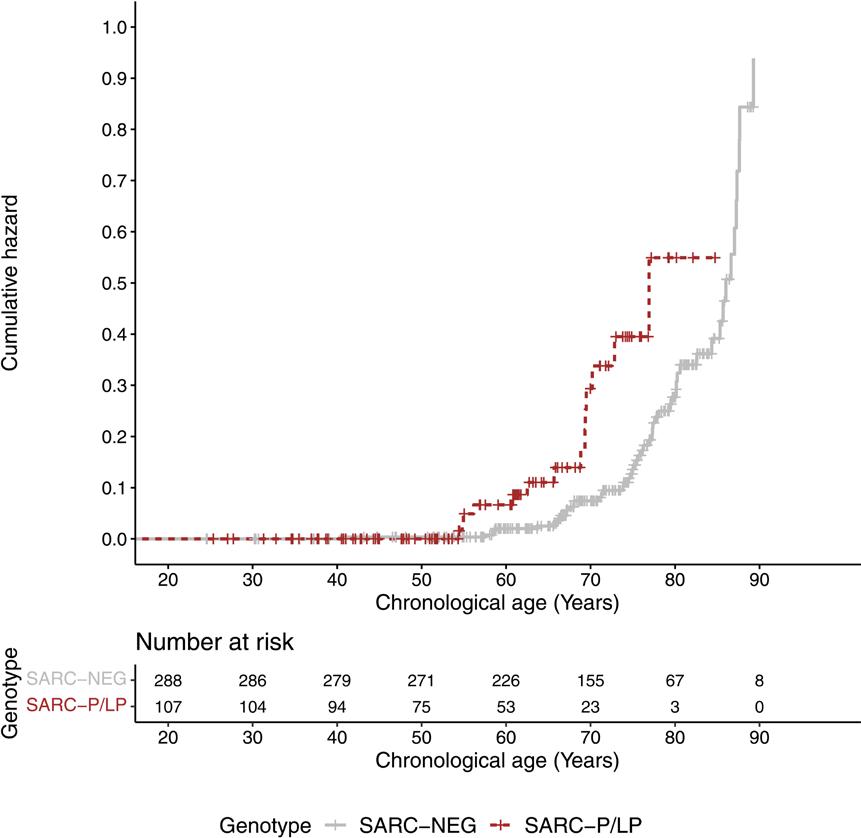
**Cumulative hazard plot.** All-cause mortality in individuals with hypertrophic cardiomyopathy (HCM) carrying pathogenic/likely pathogenic sarcomeric variants (SARC-P/LP) compared with genotype negative (SARC-NEG; hazard ratio, 2.66 [95% CI, 1.42–4.96]; *P*=0.002).

All-cause mortality was available for all 436 HCM cases and was found to be 17.4% across the entire cohort. By the end of follow-up, 14 (13.1%) of the SARC-P/LP, 7 (17.1%) of the SARC-VUS, and 55 (19.1%) of the SARC-NEG patients had died. The SARC-P/LP patients were younger at recruitment (median age, 49 years; IQR, 38–61; *P*<0.001) than both SARC-VUS (median age, 61 years; IQR, 52–67) and SARC-NEG patients (median age, 62 years; IQR, 53–71). The proportional hazards assumptions were met. In an unadjusted Cox model, SARC-P/LP patients had increased risk of death (n=395; hazard ratio, 2.66 [95% CI, 1.42–4.96]; *P*=0.002; Kaplan-Meier plot in Figure 5) relative to SARC-NEG participants and shorter life span (median age at death, 67 years; IQR, 5–70 versus 76 years; IQR, 68–84; *P*=0.0003). This relationship was also independent of genetic sex and ancestry in a multivariable analysis (Tables S9 and S10).

## DISCUSSION

HCM is characterized by phenotypic heterogeneity, which presents challenges for developing personalized profiles of dynamic disease status to guide patient management. Conventional morphological descriptions of hypertrophic remodeling have relied on 2-dimensional appearances with a diverse range of subjective shape classifications proposed with varying genotype enrichment.^14,15^ Here, we report a systematic study of 3D genotype-phenotype associations in sarcomeric and nonsarcomeric HCM and identify natural groupings of morphologically similar patients while preserving the continuous distribution of phenotypic severity, clinical risk, and enrichment for HCM-associated rare variants and common variant modifiers.

The classification of HCM morphology has relied on recognizing regional distributions and shape features of LV hypertrophy, initially with echocardiography and more recently using CMR, with between 6 and 12 different patterns described.^16–18^ Broadly, these comprise basal (sigmoid), midventricular (reverse septal curve), apical, and diffuse patterns, as well as mixed phenotypes. Within these heterogeneous endophenotypes, those with SARC-P/LP variants are more likely to have reverse septal curve morphology with fibrosis, and those who are SARC-NEG more likely to have isolated basal septal hypertrophy with obstruction but less fibrosis.^7^ Although such approaches either associate imaging phenotypes that share common features or stratify by genotype status, they do not objectively classify the spectrum of phenotypic expression that is characteristic of HCM. Advances in image analysis have allowed detailed 3D models of ventricular shape and motion to be extracted from routine CMR that possess spatial consistency within and between cohorts, allowing precise aggregation of phenotypic data in diverse populations.^5^ This enables regional genotype-phenotype associations to be made by variant status and allows data-driven approaches for unsupervised learning of complex traits.

In our study, SARC-NEG HCM had increased LV mass compared with SARC-P/LP patients but this masked differences in the patterns of hypertrophy between genotypes. The 3D genotype-phenotype mapping showed that SARC-P/LP patients had lower wall thickness across most of the LV, apart from the basal septum, when compared with patients with HCM who were SARC-NEG. Relative to healthy controls, there was diffuse hypertrophy that was most marked in the basal septum. Cross-sections of the LV show a generally smaller end-diastolic cavity in SARC-P/LP patients compared with controls, especially in the long axis direction, while SARC-NEG HCM patients have a similar cavity size to controls but show more diffuse hypertrophy. Differences in global volumes and mass between genotypes are, therefore, the net effect of opposing regional changes in the distribution and degree of hypertrophy.

To reconstruct trajectories of how HCM phenotypes transition toward differentiated states, we applied a machine learning approach using reversed graph embedding to learn branch points that define significant divergences in 3D morphology that requires no a priori information about the genes or environment that modifies disease biology.^19^ The resulting tree structure provides a 2-dimensional representation of complex phenotypic variation that can be generalized to diverse data sets,^12^ and addresses potential sources of bias and subjectivity in human visual assessment of HCM.^20^ The tree maps disease trajectories from undifferentiated states in the center to more characteristic morphologies in the distal branches. This provides a visual representation of how phenotypic variation translates to variation in genetic enrichment and survival. We also demonstrate how new patients can be mapped to a position in the tree, facilitating individualized patient stratification.

The interaction between hypertension and HCM presents significant diagnostic challenges, including when it should be considered a comorbid condition rather than the underlying cause of hypertrophy.^10^ The phenotypic branch with the most severe degree of hypertrophy (1) was enriched for controlled hypertension and comprised predominately SARC-NEG patients. The pattern of hypertrophy predominantly affects the midventricle to apex in this branch and may include the phenotypic spectrum of apical HCM.^21^ Nonsarcomeric HCM is partly an exaggerated response to diastolic hypertension in genetically susceptible individuals, and this phenotype shares features of hypertensive patients without HCM.^3,22^ Recognition of these patients’ phenotype at the point of diagnosis could help to identify nonsarcomeric HCM cases associated with modifiable risk factors. Late enhancement burden was the highest in this group, suggesting that enrichment for replacement fibrosis is associated with more severe manifestations of HCM, despite a lower prevalence of pathogenic variants, which may reflect a later stage of predominantly nonfamilial disease than other HCM clusters.^23^ The other common phenotypic branch (4) had milder hypertrophy, a low prevalence of hypertension and the most enrichment for P/LP variants though remains predominantly comprised of SARC-NEG patients. This branch was also the most enriched for a family history of HCM and may include patients earlier in the natural history of disease recruited to the registry. In common with previous registry studies, we also found P/LP variants to be predictors of mortality,^24^ and this nonobstructive HCM phenotype enriched for familial disease and sarcomeric variants had the poorest outcome with risk increasing for patients expressing the most differentiated phenotype in the tree structure. Early prediction of molecular subtypes of HCM could be of value when considering emerging therapies that act upstream of the underlying genetic cause.^25^

LV outflow tract obstruction is independently associated with adverse HCM-related outcomes,^26^ may benefit from surgical intervention, and is amenable to treatment with myosin inhibitors.^27^ It is associated with septal hypertrophy and ventricular remodeling,^28^ although it may also occur in milder cases of HCM.^29^ We found that the phenotype in branch (2) with pronounced mid-to-basal asymmetrical hypertrophy in a diffusely hypertrophic LV was strongly enriched for stress-confirmed LV outflow tract obstruction and hyperdynamic function. As this phenotype becomes more differentiated from an average HCM morphology, the survival probability decreases. The last main group (3) in the taxonomy is associated with isolated hypertrophy of the basal septum with relatively mild LV hypertrophy elsewhere. A similar benign phenotype has been recognized as part of normal aging whose appearances may overlap with HCM.^30^ We also found this phenotype to be associated with better survival and a low probability of P/LP variants.

Our approach differs from previous studies describing phenotypic patterns in HCMs as we adjust for age and sex, which affect both disease physiology and outcomes, and so the classification is independent of these risk factors.^31^ While our approach also aims to identify groups of similar morphology, it does this using a data-driven approach without human subjectivity and maintains a continuum of disease expression and risk factors across the taxonomic tree. We also identify potential causal modifiers of disease expression with common variants contributing to the differentiation and severity of phenotypes in addition to their reported role as risk markers for survival and adverse events in HCM.^32^ We have used computer vision techniques to build patient-specific cardiac models of disease that go beyond traditional imaging parameters used to stratify patients or as inputs into clinical risk models. Multiparametric imaging-based models offer more accurate prediction of composite end points in HCM compared with conventional risk factors,^33^ and machine learning models using conventional imaging parameters have better discrimination of HCM genotype than clinical scores.^34^

We offer a novel taxonomy of the complex genotype-phenotype architecture of HCM, with potential for clinical translation to precision models, which utilize multiparametric data in an agnostic way, without reliance on a priori clinical assumptions or labels. We also demonstrate how unseen external data can be readily mapped to coordinates in the tree to explore personal dynamic risk profiles. This new classification may also provide a basis for further mechanistic exploration, through integration of multiomic profiles or trial design comparing discovered subgroups. Such feature extraction from imaging could play a role in improving patient stratification as automated analysis in HCM becomes more widely available but will require a standards-based approach for multicohort data integration.

This study has limitations. The HCM registry recruited consecutive patients with a clinical diagnosis from a cardiomyopathy clinic or undergoing CMR and so may be less enriched for symptomatic or more severe phenotypes compared with other cohorts. This reflects the spectrum of disease seen in clinical practice but may account for the relatively low event rate observed. Most of the development cohort was European, although we found that the mapping could generalize well to other ancestries. This provides a conservative estimate of generalizability, and some external patients with HCM had phenotypic features that may be better represented by training a model on more diverse multiancestry data. We used CMR as this is regarded as the gold standard for phenotyping, but it is less widely available than echocardiography.

In summary, we provide a data-driven taxonomy for understanding dynamic phenotypic diversity in HCM that reflects a continuum of disease, genetic risk, and outcomes. A systematic representation of HCM diversity onto which new patients can be mapped has the potential to enable more individualized assessment, stratification, and treatment strategies.

## ARTICLE INFORMATION

### Acknowledgments

We thank Drs Tim Dawes and Carlo Biffi (Imperial College London) for contributing to the initial development of the 3-dimensional cardiac modeling. We also acknowledge Dr Jinming Duan (University of Birmingham) in the initial development of the segmentation pipeline. L. Curran and Dr Marvao performed data curation and analysis, and drafted the article; Dr Inglese, Dr Clement, and P.-R. Schiratti performed analysis and visualization of the data; Dr McGurk, R.J. Buchan, and P. Theotokis performed the genetic analysis; S. Li, Dr Jafari, and Dr Bai performed the image analysis; M. Shah and S.L. Zheng performed formal analysis of the data; Drs Raphael, Baksi, Pantazis, and Halliday collected clinical data in the United Kingdom; Drs Pua and Chin collected clinical and genetic data in Singapore; Drs Pennel, Cook, Prasad, Jurgens, Tadros, Bezzina, and Watkins provided clinical or genetic resources for the study; Dr Ware supervised the genetic analyses; Dr O’Regan conceived the project, provided supervision, and provided funding. All authors contributed to the article and approved the submitted version. D O’Regan, J Ware, H Watkins and S Cook are supported by CureHeart, the British Heart Foundation’s Big Beat Challenge award (BBC/F/21/220106).

### Sources of Funding

The study was supported by the Medical Research Council (MC_UP_1605/13); National Institute for Health Research Imperial Biomedical Research Center and Royal Brompton Cardiovascular Biomedical Research Unit; British Heart Foundation (RG/19/6/34387, RE/18/4/34215, FS/IPBSRF/22/27059, and FS/ICRF/21/26019); Engineering and Physical Sciences Research Council (EP/W01842X/1); Academy of Medical Sciences (SGL015/1006); Mason Medical Research Trust; Sir Jules Thorn Charitable Trust (21JTA); National Heart and Lung Institute Foundation Royston Center for Cardiomyopathy Research; and the Rosetrees Trust. For the purpose of open access, the authors have applied a creative commons attribution (CCBY) license to any author-accepted article version arising.

### Disclosures

Dr Ware has consulted for MyoKardia, Inc, Foresite Labs, and Pfizer and receives research support from Bristol Myers Squibb outside the submitted work. Dr O’Regan has consulted for Bayer AG and Bristol Myers Squibb and also receives research support from Bayer AG outside the submitted work. Dr Halliday is on an advisory board for AstraZeneca. The other authors report no conflicts.

### Supplemental Material

Supplemental Methods

Tables S1–S11

Figures S1–S11

References 35–67

## Supplementary Material
