# Supplementary material for: Genotype-Phenotype Taxonomy of Hypertrophic Cardiomyopathy
Source: Circ Genom Precis Med. 2023 Nov 28;16(6):e004200. doi: 10.1161/CIRCGEN.123.004200 (PMC10729901; doi:10.1161/CIRCGEN.123.004200)
Supplement: Supplementary file 1 [file hcg-16-e004200-s001.pdf]

Supplemental Material

HCM participants

In total 710 patients with a clinical diagnosis of HCM, either seen in the inherited cardiomyopathy service or referred for CMR imaging, were consecutively enrolled into a prospective registry at the National Institute for Health Research (NIHR) Royal Brompton Hospital Cardiovascular Biobank project between 2009-2015, of whom 436 were included in this study. All participants provided written informed consent and the study was approved by the National Research Ethics Service (19/SC/0257). HCM diagnosis was independently adjudicated by a cardiomyopathy specialist based on established clinical and CMR criteria where all patients met the American Heart Association criteria for diagnosis.[10] This was defined as a wall thickness of 15mm or greater, or 13–14mm if there was a first degree relative with HCM, not explained by another cardiac or systemic disease-causing abnormal loading conditions, or had disproportionate apical wall thickness and tapering in keeping with an apical HCM phenotype.[35]

Patients were excluded from analysis based on age (< 16 years at time of CMR), missing demographic or clinical data, contraindication to CMR, previous history of septal ablation, cardiac transplantation or myectomy at baseline. A history of hypertension or diabetes was documented, as well as current medication at time of enrolment to the study. The cohort underwent detailed clinical, imaging and genetic assessment. All patients underwent CMR for assessment of cardiac chamber volumes and function (1.5T, Siemens Sonata or Avanto, Siemens Medical Systems, Erlangen, Germany). Variables reported were collected at enrolment to the study. The CMRs selected for analysis were those closest to the date of enrolment or the first diagnostic study available. Where present, left ventricular outflow tract obstruction (LVOTO) was confirmed through stress echocardiography. Unrelated Singaporean patients with a diagnosis of HCM (n = 60) were prospectively recruited from the National Heart Centre Singapore. Patients gave written informed consent to participate which was approved by the Singhealth Centralised Institutional Review Board (2020/2353) and Singhealth Biobank Research Scientific Advisory Executive Committee (SBRSA 2019/001v1). Singaporean subjects underwent an equivalent CMR protocol at 1.5T (Aera, Siemens, Erlangen, Germany) or 3T (Ingenia, Philips, Best, Netherlands).

Conventional CMR analysis was undertaken by accredited operators using semi-automated software (CMRtools, Cardiovascular Imaging Solutions, London, UK).

UK Biobank participants

The UKB study recruited 500,000 participants aged 40 to 69 years old from across the United Kingdom between 2006 and 2010 (National Research Ethics Service, 11/NW/0382).[36] This study was conducted under terms of access approval number 40616. In each case, written informed consent was provided.

A sub-study of UKB invited participants for CMR for assessment of cardiac chamber volumes and function using a standard protocol (1.5T, Siemens Aera, Siemens Medical Systems, Erlangen, Germany).[37] As a reference population, we selected 16,691 participants that did not meet criteria for left ventricular hypertrophy and were classified as genotype negative (SARC-NEG) by having no variants in genes that may cause or mimic HCM (see Sequencing and variant categorisation).

Cardiac phenotyping using machine learning

Segmentation of the cine images in both UKB and HCM groups was performed using a deep learning neural network algorithm developed and optimised in-house. The performance of image annotation using this algorithm is equivalent to a consensus of expert human readers and achieves sub-pixel accuracy for cardiac segmentation.[38] The label maps were super-resolved and registered to a cardiac atlas enabling consistent quantitative three-dimensional phenotypic analysis within and between patient groups.[39]

Myocardial wall thickness was measured along radial line segments connecting the endocardial and epicardial surfaces perpendicular to the myocardial centreline and excluding trabeculae. Chamber volumes and mass were calculated from the segmentations according to standard post-processing guidelines.[40] Myocardial strain analysis was performed using non-rigid free-form deformation image registration.[41] Trabecular traits were quantified using fractal dimension (FD) analysis where a higher value indicates more complex trabeculation.[42]

Sequencing and variant categorisation

Panel sequencing was completed in the HCM patient cohort, as previously described.[43] The patients were sequenced using either a custom SureSelect capture panel targeting genes associated with inherited cardiac conditions or the Illumina TruSight Cardio panel. Sequencing was performed on either the SOLiD 5500xl platform, or the Illumina HiSeq, MiSeq or NextSeq platforms. The Singaporean cohort underwent targeted genetic sequencing using the TruSight Cardio panel and an equivalent pipeline as previously reported.[44]

Patients were divided into three genetic strata. Patients carrying at least one potentially-causative rare variant (allele frequency <0.00004)[45] in any of 8 sarcomere-encoding genes robustly associated with HCM were considered genotype positive. These were further stratified into (i) those carrying variants previously confidently classified as pathogenic / likely pathogenic (SARC-P/LP) in ClinVar, and confirmed on our review, or else curated as P/LP according to ACMG criteria using

the semi-automated CardioClassifier decision support tool[46] (n = 107), as previously published,[5]; and (ii) those carrying sarcomeric variants of uncertain significance (SARC-VUS), comprising variants in the same 8 genes, that are consistent with known disease mechanisms and sufficiently rare, but with insufficient evidence to classify robustly as P/LP.[47,48]

Individuals were classified as genotype negative (SARC-NEG) if they had no rare protein-altering variant (minor allele frequency <0.001 in the UKB and the Genome Aggregation Database)[49] in any of 25 genes that potentially cause HCM (definitive or moderate evidence according to international curation)[50] or cause syndromes that can present with isolated left ventricular hypertrophy (genocopies).[50] In order to generate the most robust set of true genotype negatives, individuals carrying a protein-altering variant in any these 25 genes, but that was not sufficiently rare to be considered potentially causative of monogenic HCM was excluded from the analysis. Further details are given in Supplementary Materials. Common genetic variation contributes substantially to HCM risk and we also assessed the relationship between phenotype and polygenic score (PGS) derived from a case-control HCM genome wide association study (GWAS) in the 100,000 Genomes Project.[32]

Variant curation pipeline

This pipeline can be found on GitHub (https://github.com/ImperialCollegeLondon/HCM-taxonomy) and has been previously published.[5] All genetic data was annotated using Ensembl Variant Effect Predictor (VEP; version 105)[51] with plugins for NMD, SpliceAI (version 1.3.1),[52] ClinVar (version 2022 01 15),[53] gnomAD (version r2.1),[49] and LOFTEE.[49] The VEP output was analysed using R (version 3.6.0).

Protein-altering variants, defined using MANE transcripts, that had a MAF of <0.1% in gnomAD for variants identified in cases, and <0.1% in gnomAD and UK Biobank for variants identified in the UK Biobank, were included in the analyses. Protein altering variants were specified as high or moderate impact by Sequence Ontology and Ensembl, with the addition of splice region variants for further curation. The variants were filtered for genes and protein consequences of interest,[50] to include 8 definitive-evidence sarcomeric HCM genes (*MYH7, MYBPC3, MYL2, MYL3, ACTC1, TNNI3, TNNT2, TPM1*), 3 medium-evidence HCM genes (*CSRP3, TNNC1, JPH2*), 2 intrinsic cardiomyopathy genes (*ACTN2* (moderate classification), *PLN* (definitive classification)), and 12 syndromic genes that can cause isolated left ventricular hypertrophy (*FHL1, TTR, FLNC, GLA, LAMP2, PRKAG2, PTPN11, RAF1, RIT1, ALPK3, CACNA1C, DES*). *FLNC, ALPK3, ABCC9, CRYAB, MYO6, and RIT1*, were not sequenced in cases, but were analysed in UK biobank. No protein-altering variants were identified in *TNNC1* in the case cohort.

Splice region variants (outside the canonical splice donor and acceptor sites) were assessed in two ways; i) via ClinVar report: splice region variants found pathogenic with at least 2 star evidence for HCM in ClinVar and reported functional evidence for splicing were termed "splice confirmed"; if the functional evidence was unclear the protein consequence remained unchanged; if there was functional evidence of an alternative mechanism to splicing, the protein consequence was renamed (e.g. missense variant); ii) via prediction threshold: of the splice region variants, they were excluded if they did not meet the spliceAI threshold of >0.8, and these thresholds were used to identify potentially splice-causing variants of those splice region variants identified with a non-synonymous consequence flag (e.g. intron variant).

The pipeline then consisted of three main filtering steps which resulted in an output of four columns of binary code flagging genotype status (heterozygous, compound heterozygotes, and homozygotes, combined) as "1": SARC-NEG – Individuals who do not harbor any rare non-synonymous variants in any of the 25 genes of interest. This was a stringent filter to identify an unambiguous genotype-negative control group.

SARC-VUS – Individuals harboring rare variants in one or more of the 8 definitive HCM-associated sarcomere-encoding genes. Rare variants were restricted to known disease-associated variant classes. This step separated the variants into two subsets: i) Loss of function (LoF) alleles (group A), which contained only the gene *MYBPC3*, and filters for the protein consequences of stop gained, splice acceptor variant, splice donor variant, frameshift variant, and splice region variant (with additional in silico evidence of an effect on splicing). LOFTEE was incorporated in this step to exclude loss of function (LoF) variants that were flagged as "low confidence" (LC) and other LOFTEE flags, such as "NAGNAG sit" requiring reannotation to non-LoF variant status; ii) Protein altering (PAV) alleles (group B), which included all 8 sarcomeric genes, including *MYBPC3*, and filters for the protein consequences of missense variant, inframe insertion, and inframe deletion.

Both groups included additional positional annotation (LoF variants found in the last exon or 55bp into the penultimate exon or stop gained variants with a NMD flag using the NMD plugin), this included variants that introduce a protein-truncating variant (PTC) and predicted to lead to nonsense-mediated decay (NMD). The variants flagged 'coding sequence variant' and 'protein altering variant' were manually curated, as were 'stop lost' and 'start lost' which were examined via ENSEMBL sequence and UCSC Genome Browser to identify in-frame rescues nearby. To be included in the SARC VUS group, the variants were required to meet a maximum gnomAD filter allele frequency (FAF) threshold for HCM (<0.00004) and excluded variants deemed P/LP for DCM on ClinVar.

SARC-P/LP is as SARC-VUS, plus annotated as P/LP according to the ACMG guidelines.[48] Variants were reviewed if reported as P/LP for HCM by at least one submitter in ClinVar, or if flagged as P/LP by the CardioClassifier decision support software.[46] Variants that did not meet either of these criteria were not individually reviewed.

Outcome measures

Data were collected to measure all-cause mortality in the HCM cohort. Outcomes were verified through search of the NHS Shared Care Records. Patients were followed up for a median of 10.2 years from date of study enrollment.

Statistical analysis and data modelling

Statistical analysis was performed with R (version 4.0.3) and RStudio Server (version 1.2; Boston, MA), unless otherwise stated. Variables were expressed as percentages if categorical, mean ± standard deviation (SD) if continuous and normal, and median ± inter-quartile range (IQR) if continuous and non-normal. Baseline anthropometric data were compared by Kruskal-Wallis tests and, if differences were identified, a Wilcoxon test was used for pairwise comparisons with Benjamini-Hochberg adjustment for multiple testing. Clustering of clinical data from HCM patients was performed using UMAP (uniform manifold approximation and projection)[54] for dimensionality reduction followed by a K-means algorithm evaluated with a silhouette score to find the optimal number of clusters.

The association between genotype and three-dimensional phenotype was assessed by fitting univariable regression models at each vertex of the cardiac mesh, controlling for false discovery, with corrected beta coefficients plotted on the epicardial surface.[5,55] Clustering of subjects by their 3D left ventricular wall thickness, adjusted for age, sex and ancestry, was performed through partitioning the shared nearest neighbour (SNN) graph[56] with the multilevel refinement Leiden algorithm.[57] The SNN graph and its partitions were determined using the functions available in `Seurat`.[58] The clusters were visually inspected by UMAP projection and their stability was assessed through bootstrapping using `fpc`. We used DDRTree (discriminative dimensionality reduction via learning a tree) to project the 3D left ventricular wall thickness into a 2D tree structure to visualise the distribution of HCM phenotypes.[12,59]

The predictive power of the DDRTree mapping for P/LP genotype was tested with a generalized additive model (GAM) fitted on the tree coordinates, using a 10-fold cross validation repeated 3 times. Survival probability to median observed age was estimated with a Cox proportional hazards model fitted on the cubic spline of tree coordinates of each individual and their relative position in the tree. Association between the DDRTree mapping and polygenic risk score was assessed with a logistic regression GAM model using the subjects' coordinates as independent variables and the binarized polygenic risk score (thresholded at median) as outcome. The predictions were obtained using a 10-fold cross validation.

The primary survival analysis was performed in individuals from the HCM cohort with chronological age as time-scale and adjusting for genetic sex and ancestry (dichotomised by white European ancestry). Participants with SARC-P/LP variants were compared with pooled participants without variants and with SARC-VUS carriers. Proportional hazards assumption as assessed using Schoenfeld residuals was not violated.

Estimation of wall thickness from meshes

Three dimensional meshes with the local wall thicknesses (WT) were generated from the myocardial segmentations of the end systolic (ES) and end diastolic (ED) phases, using previously published methods.[55,60,61] In order to make the modelling of the WT computationally tractable, the meshes were decimated by 99%. Specifically, because of the one-to-one correspondence between subjects' meshes vertices and atlas vertices, the decimation was applied to the atlas only, and the closest resulting vertices to the original atlas were selected from all meshes. This allowed us to preserve the correspondence between the individual vertices across all meshes.

Unsupervised analysis of wall thickness values

Wall thicknesses were firstly adjusted by age at cardiac magnetic resonance (CMR) imaging, sex and ancestry using a linear regression, and normalized afterwards using the `Seurat` package for R.[58] To remove the intrinsic correlations between the values, due to the spatial nature of the data, and to reduce the effect of statistical noise, the data was compressed applying a principal components analysis (PCA), and retrieving the first 50 principal components.

A shared nearest neighbor graph (SNN) was built from the compressed data, where the nodes corresponded to the subjects, and the edges corresponded to the Jaccard index between the nearest neighbours of each pair of subjects. The nearest neighbors corresponded to the 20 subjects with the smallest cosine distance from each subject. Thus, the SNN graph was partitioned using the multilevel Louvain algorithm, with resolutions varying from 0.1 to 1 in steps of 0.1. Both SNN and its partitioning were performed using `Seurat` package for R.[58]

The optimal resolution was chosen by inspecting the cluster stability with `clustree`, corresponding to the partitioning before any mixing of cluster assignment was visible[62] (Supplementary Figs. II, III). Additionally, stability of the clusters was assessed by rerunning the clustering on 1000 random subsets, each with a size equal to 80% of the whole cohort, using the function `clusterboot` from the `fpc` package for R.

If more than one partitioning was found corresponding to the same branch structure of the `clustree` plot, that with the greater stability was chosen (Supplementary Tables I, II).

DDTree modelling of wall thickness values

DDRTree was applied on the adjusted wall thicknesses for age at CMR, sex and ancestry. Following the same approach described in the previous section, the first 50 principal components were calculated and used as input for the DDRTree algorithm.[19] All parameters were kept as default. The underlying tree structure, the result of the procedure, was automatically partitioned into branches by considering as branching those points with a degree equal to 3. Subsequently, we merged small branches into their larger neighbour, such that the main structure of the tree was preserved. This step aimed at reducing the number of phenotypic sub-types and increasing the statistical power of the subsequent modelling. Firstly, branches consisting of less than 5 individuals were merged to the closest connected branch. Subsequently, short terminal branches, with a geodesic length smaller than 5% of the graph length were merged with their parent with respect to the center of the tree, as they represented low-diversity individuals. The branches connected to these were then merged, as bifurcation no longer existed (Supplementary Fig. VIII- IX).

A set of clinical and anthropometric measures were tested for statistical association with the tree branches. We first applied a Kruskal Wallis test to continuous variables and χ^2 test to discrete variables. Those variables showing a significant association (Benjamini-Hochberg adjusted $P < 0.05$) were post-hoc tested. A Dunn test was used to test each pair of branches, whereas the exact Fisher test was used for discrete variables in one-vs-all fashion. All *P*-values were adjusted for multiple testing using Benjamini-Hochberg method.

Finally, we tested the consistency of the tree structure after removing 8 related subjects from our original analysis. The association between the tree's main axes and 1) genotype status, and 2) PRS remained unchanged (Supplementary Table V-VI). Additionally, the relative position of the subjects was consistent between the two trees, as shown by the high value of the correlation between the pseudotime of the two models (Spearman's rho = 0.89, Supplementary Fig. X).

External validation

Wall thicknesses were residualized by linear regression using sex and age at scan as covariates. In this way, we could compare the similarities between the intra-cohort statistical properties of WT values. The first 5 principal components were estimated from the development cohort and used to project the adjusted Singaporean WT values. The development cohort principal component scores were used to fit two random forest models aimed at predicting the x and y tree coordinates.

Performances of the models were evaluated using a 10-fold cross-validation, repeated 3 times. The fitted models were therefore used to predict the tree coordinates of the Singaporean individuals, from their principal component scores. Faithfulness of the tree mapping for the Singaporean cohort was evaluated by the "trustworthiness" M_1 measure, which estimates how observations that are similar in the original high dimensional space are placed close to each other in the low dimensional space. It ranges from 0 to 1, with larger values corresponding to a better representative low dimensional mapping.[13]

In order to evaluate the consistency of the local statistical patterns between the development tree and the projected Singaporean individuals, we considered Spearman's correlation between nearest neighbour points in the tree. We estimated the correlations between the adjusted WT of the closest points in the development tree, and compared their distribution with that of the correlations between the Singaporean WT and their closest points in the development tree. In order to test differences in the distributions, we used a Wilcoxon test for difference of medians, and a Kolmogorov Smirnov test for difference of distributions.

Supplementary Tables I- XI and Figures I-XI

Supplementary Table I. Cluster stability at end diastole. Results from the analysis of the cluster stability for end diastolic wall thickness (ED WT) in 1000 subsets. The possible values range between 0 and 1, where 0 means that the cluster is not stable, and 1 that the clusters are identical in all repetitions. Between a resolution of 0.4 and 0.5, determined from the `clustree` plot (Supplementary Fig. II). We chose the resolution of 0.5 because it is also characterized by more stable clusters. Cluster 2 is found as an intermediate state between cluster 0 and 1, and it is less stable than the other two.

resolution	cluster 0	cluster 1	cluster 2
0.4	0.95	0.85	0.50
**0.5**	**0.95**	**0.92**	**0.78**

Supplementary Table II. Cluster stability at end systole. Results from the analysis of the cluster stability for end systolic wall thickness (ES WT) WT in 1000 subsets. The possible values range between 0 and 1, where 0 means that the cluster is not stable, and 1 that the clusters are identical in all repetitions. Resolutions from 0.1 to 0.6 are determined from the `clustree` plot (Supplementary Fig. III). The resolution of 0.1 was chosen because it is the lowest value among those with more stable clusters.

resolution	cluster 0	cluster 1
**0.1**	**0.96**	**0.96**
0.2	0.96	0.96
0.3	0.96	0.96
0.4	0.96	0.96
0.5	0.96	0.96
0.6	0.94	0.95

Supplementary Table III. Pathogenic/likely pathogenic variant prediction from tree coordinates. Fitted parameters for the GAM model used to predict individuals with P/LP variants using the 2 tree coordinates. [1]OR, Odds ratio; CI, Confidence interval

	**ED**			**ES**		
**Characteristic**	**log(OR)[1]**	**95% CI[1]**	**p-value**	**log(OR)[1]**	**95% CI[1]**	**p-value**
(Intercept)	-1.1	-1.4, -0.93	<0.001	-1.2	-1.4, -0.96	<0.001
s(Z1)			0.008			0.013
s(Z2)			0.2			0.062

Supplementary Table IV. Singaporean branch assignment. Singaporean HCM patients were assigned to the tree branches of their nearest neighbours in the development tree. In both end diastole (ED) and end systole (ES), most of the individuals were assigned to branches 1 and 4.

**Branch**	**ED**	**ES**
1	23	21
2	13	1
3	4	2
4	20	35
5	-	1

Supplementary Table V. Association between ES tree coordinates and genotype status. Coefficients of the logistic regression between the ES tree coordinates and the genotype status (genotype == "P/LP"). The results for the whole cohort tree (left) and the unrelated subjects tree are consistent. *1*OR = Odds Ratio, CI = Confidence Interval

	**Full cohort**			**Reduced cohort**		
**Characteristic**	**OR[1]**	**95% CI[1]**	**p-value**	**OR[1]**	**95% CI[1]**	**p-value**
(Intercept)	0.31	0.25, 0.39	<0.001	0.31	0.24, 0.38	<0.001
Z1	1.04	1.01, 1.06	<0.001	1.04	1.02, 1.06	<0.001
Z2	1.02	0.94, 1.11	0.6	0.99	0.91, 1.08	0.8

Supplementary Table VI. Association between ES tree coordinates and PRS. Coefficients of the logistic regression between the ES tree coordinates and PRS (PRS == "high"). The results for the whole cohort tree (left) and the unrelated subjects tree are consistent. *1*OR = Odds Ratio, CI = Confidence Interval

	**Full cohort**			**Reduced cohort**		
**Characteristic**	**OR[1]**	**95% CI[1]**	**p-value**	**OR[1]**	**95% CI[1]**	**p-value**
(Intercept)	0.99	0.78, 1.25	>0.9	0.96	0.76, 1.22	0.8
Z1	1.00	0.98, 1.02	>0.9	1.00	0.98, 1.03	0.8
Z2	1.14	1.05, 1.25	0.002	1.12	1.03, 1.22	0.012

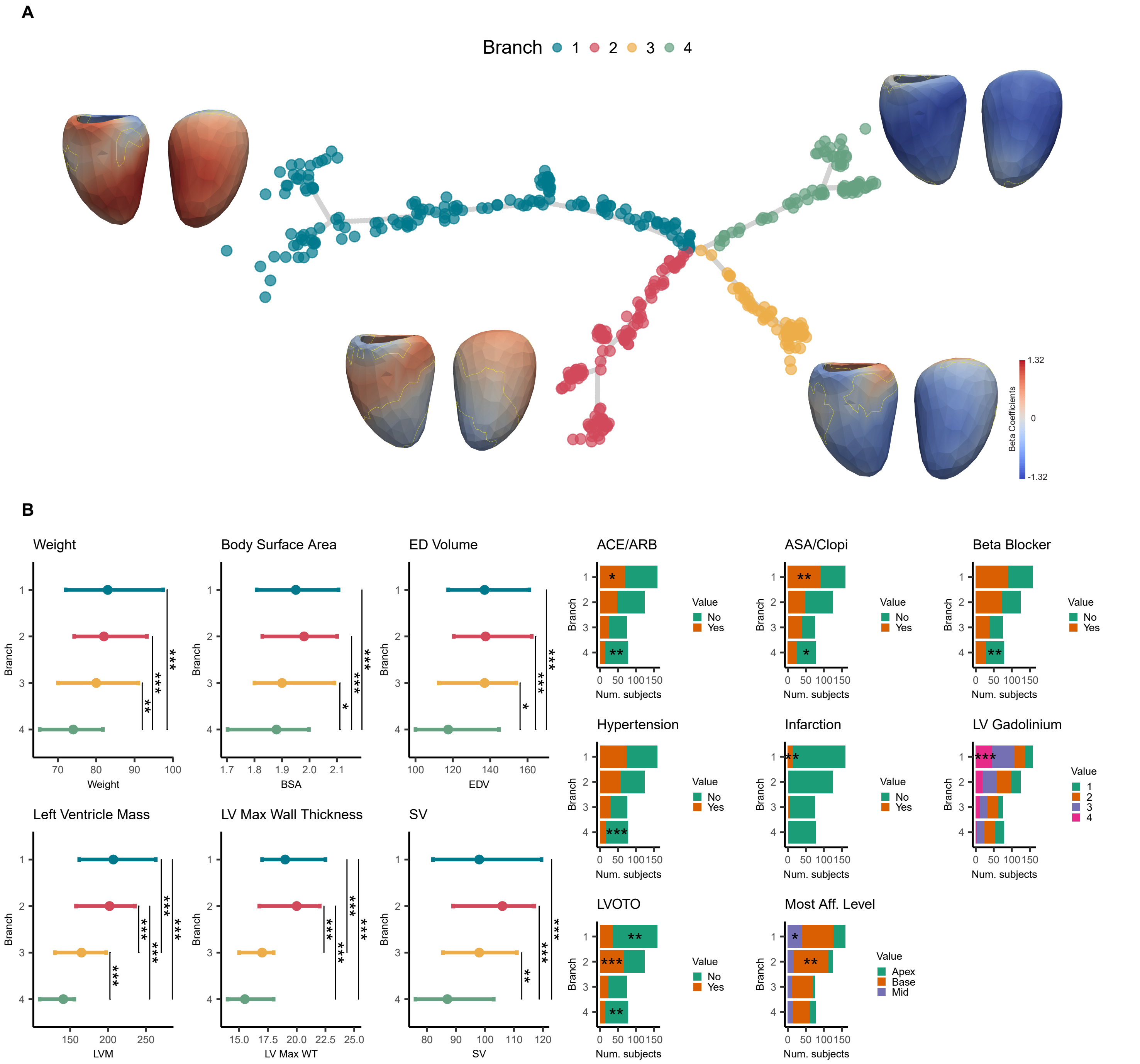

Supplementary Figure I. Phenotypic tree from 3D end-diastolic wall thickness. a. The projection of patients' 3D end-diastolic (ED) wall thickness (WT) by the DDRTree dimensionality reduction reveal the presence of four main branches that are associated to specific morphological changes of the myocardium. Each branch is represented by the decimated ED atlas mesh, coloured accordingly to the beta coefficients resulting from testing the average difference between each branch individual and the other subjects. The yellow contour denotes the areas with a beta significantly different from zero. **b.** The continuous and discrete phenotypic variables found to be significantly associated to at least one branch. For left ventricular (LV) Gadolinium, labels are as follows: 1: None, 2: Minimal, 3: Moderate and 4: Severe. The significance for the enrichment of discrete variables is reported within the bars. ACE, Angiotensin-converting enzyme inhibitors; Aff, affected; ARB, Angiotensin receptor blockers; ASA, aspirin; Clopi, clopidogrel; LVOTO, Left ventricular outflow tract obstruction; SV, stroke volume. Only the significant pairs are reported with the symbols: $* \; P \leq 0.05; ** \; P \leq 0.01; *** \; P \leq 0.001; **** \; P \leq 0.0001, n = 436.$

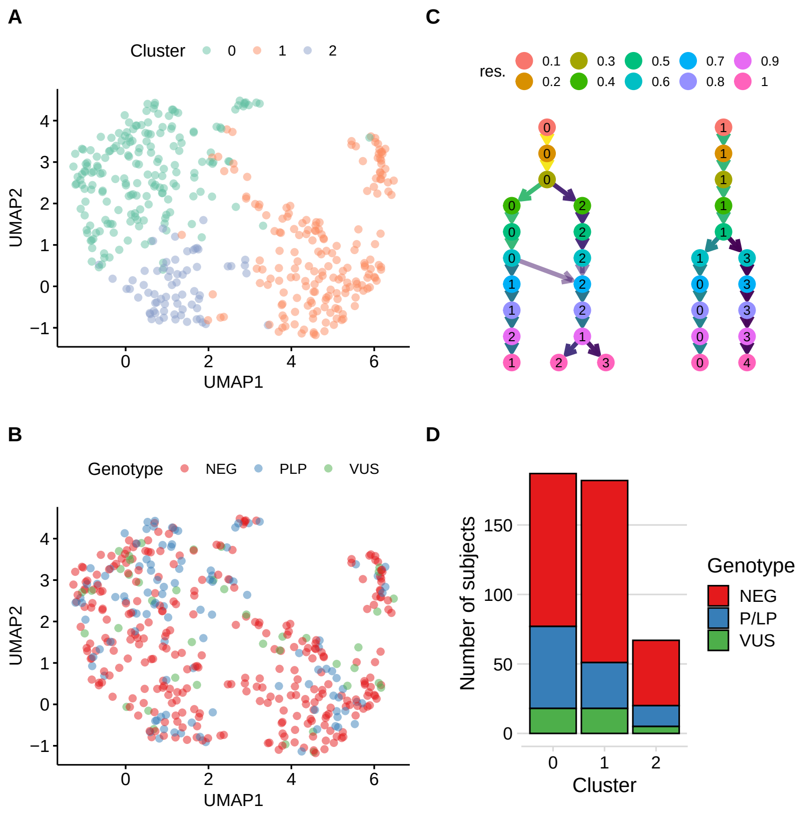

Supplementary Figure II. Selection of optimal resolution for clustering of end diastolic wall thickness. The optimal resolution for the Louvain partitioning is found by inspecting the `clustree` plot (**c.**). In this case, the value of 0.5 was chosen, corresponding to the resolution with stable branching before any assignment mixing (diagonal arrows), and the largest bootstrapping stability (Supplementary Table I). The UMAP projections in **a.** and **b.** show the parallelism between the clusters and the genotypes. **d.** Genotype proportions by cluster.

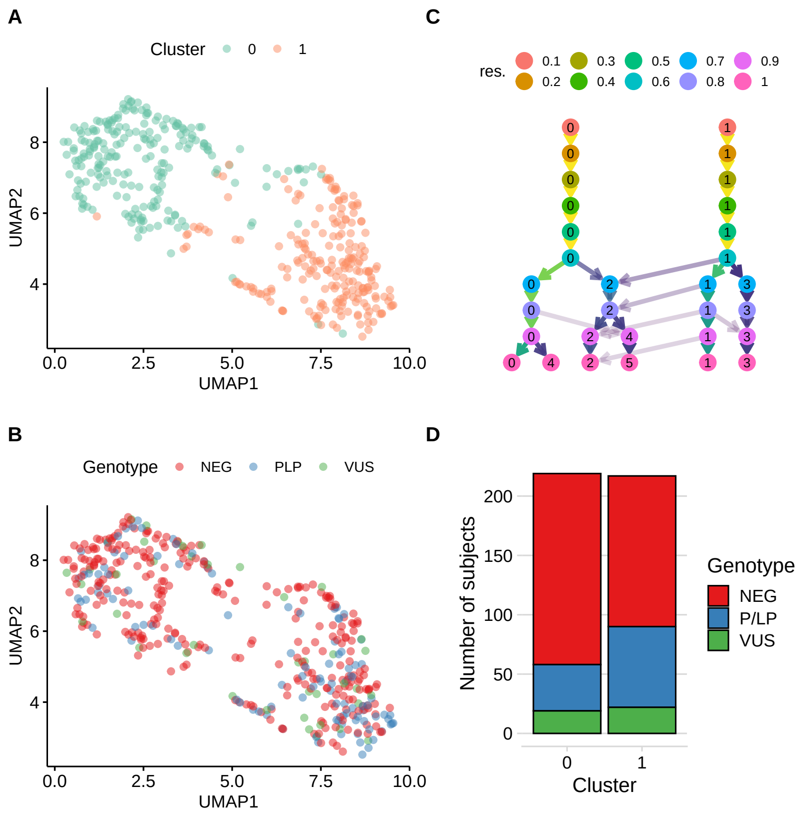

Supplementary Figure III. Selection of optimal resolution for clustering of end systolic wall thickness. The optimal resolution for the Louvain partitioning is found by inspecting the `clustree` plot (**c.**). In this case, the value of 0.1 was chosen, corresponding to the resolution with stable branching before any assignment mixing (diagonal arrows), and the largest bootstrapping stability (Supplementary Table II). The UMAP projections in **a.** and **b.** show the parallelism between the clusters and the genotypes. **d.** Genotype proportions by cluster.

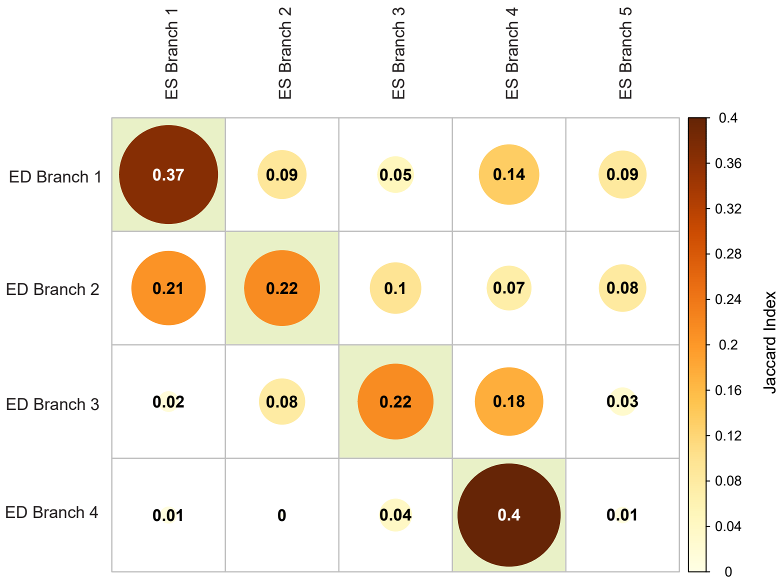

Supplementary Figure IV. Co-occurrence between end diastolic and end systolic tree branches. The Jaccard index of the subjects membership for the branches in the DDRTree from ED and ES WT shows that branches 1 to 4 have the largest co-occurrence and they can be considered capturing a similar phenotypic subpopulation. Branch 5 in end systolic DDRTree is not found in the end diastolic DDRTree and consists of an average sub-type of the cohort.

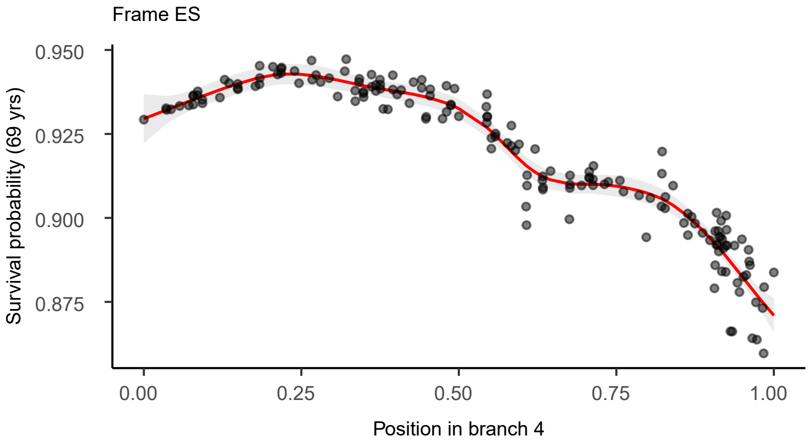

Supplementary Figure V. Survival probability in end systolic branch 4. The more distal regions of branch 4 correspond to lower probability of survival at a chronological age of 69 years. The OR between the distal and base points of the branch is 0.9773.

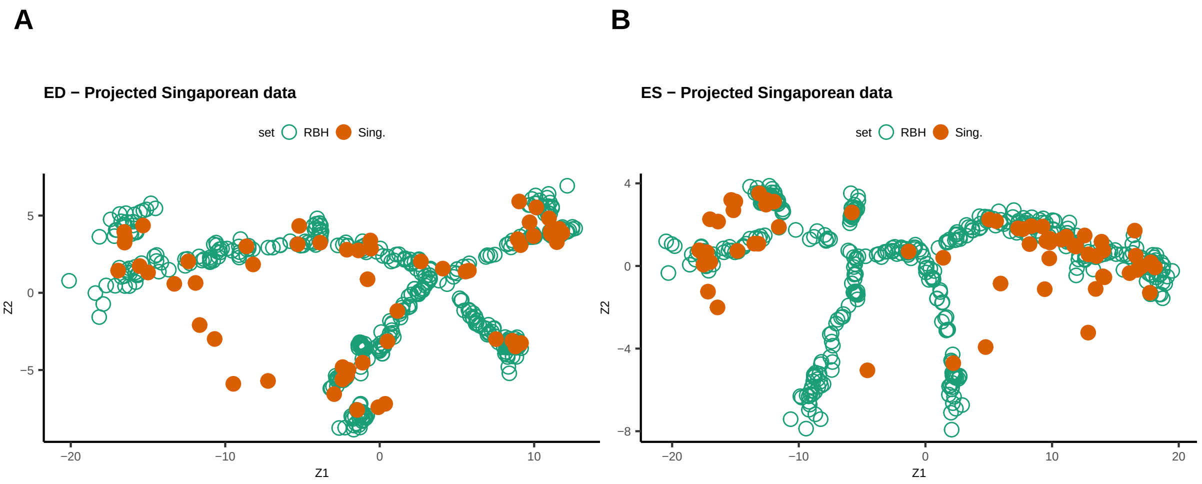

Supplementary Figure VI. Predicted tree coordinates for the Singaporean cohort. The coordinates predicted by the two random forest models for the Singaporean cohort follow the original spatial distribution of the development cohort, with few points falling outside the main structure of the tree, in both end diastole (**a.**) and end systole (**b.**). Sing, Singaporean patients; RBH, Royal Brompton Hospital patients.

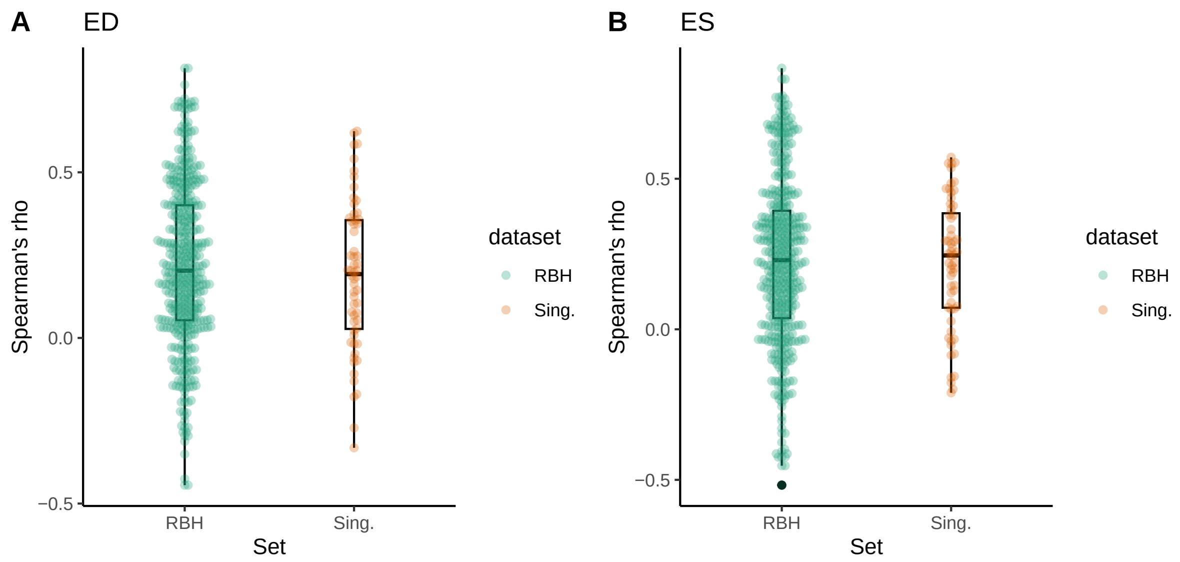

Supplementary Figure VII. Similarity between nearest tree points. Spearman’s correlation between the adjusted wall thickness of the nearest RBH points in the tree follow the same distribution of the nearest Singaporean and RBH points, in both end diastole (**a.**) and end systole (**b.**). Sing, Singaporean patients; RBH, Royal Brompton Hospital patients.

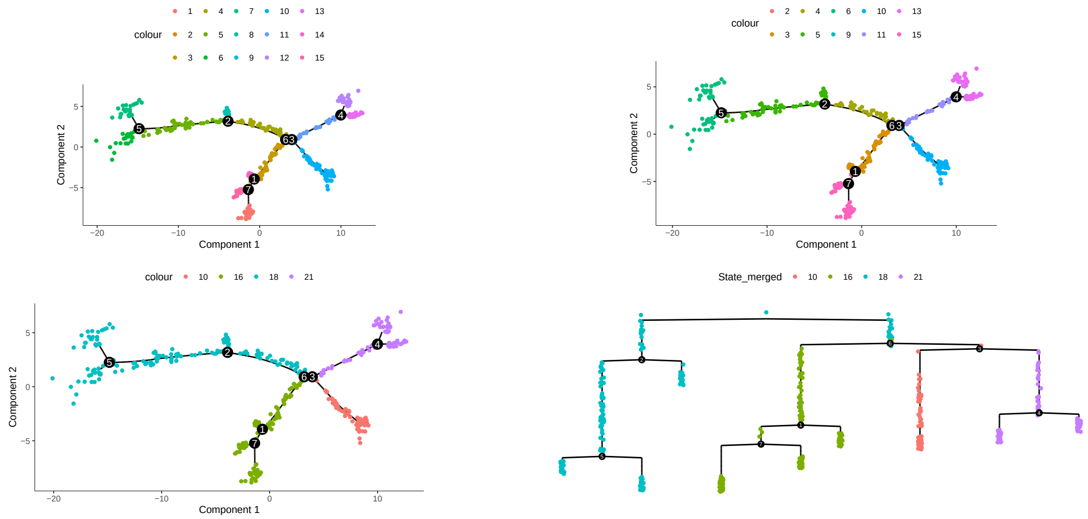

Supplementary Figure VIII. Branch merging for ED phase. Intermediate results of the branch merging process for the ED phase data. The graph at the top-left shows the branch labels determined by 'monocle' that were progressively merged: a) short leaf branches, b) branches that did not have bifurcation into two different states. The final branches qualitatively correlate with the hierarchical structure of the tree, where the root state correspond to the center of the tree.

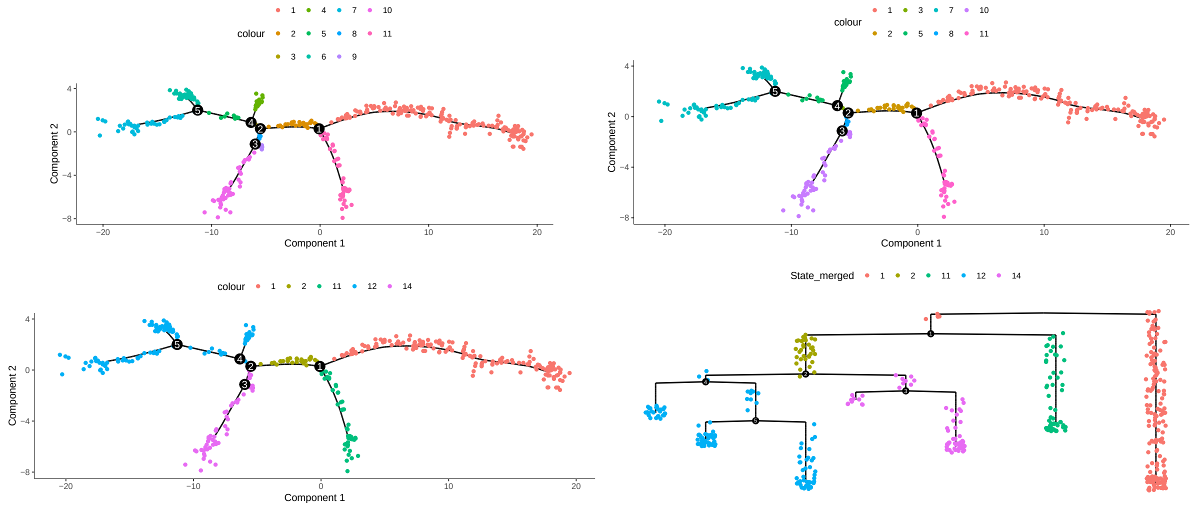

Supplementary Figure IX. Branch merging for ES phase. Intermediate results of the branch merging process for the ES phase data. The graph at the top-left shows the branch labels determined by 'monocle' that were progressively merged: a) short leaf branches, b) branches that did not have bifurcation into two different states. The final branches qualitatively correlate with the hierarchical structure of the tree, where the root state correspond to the center of the tree.

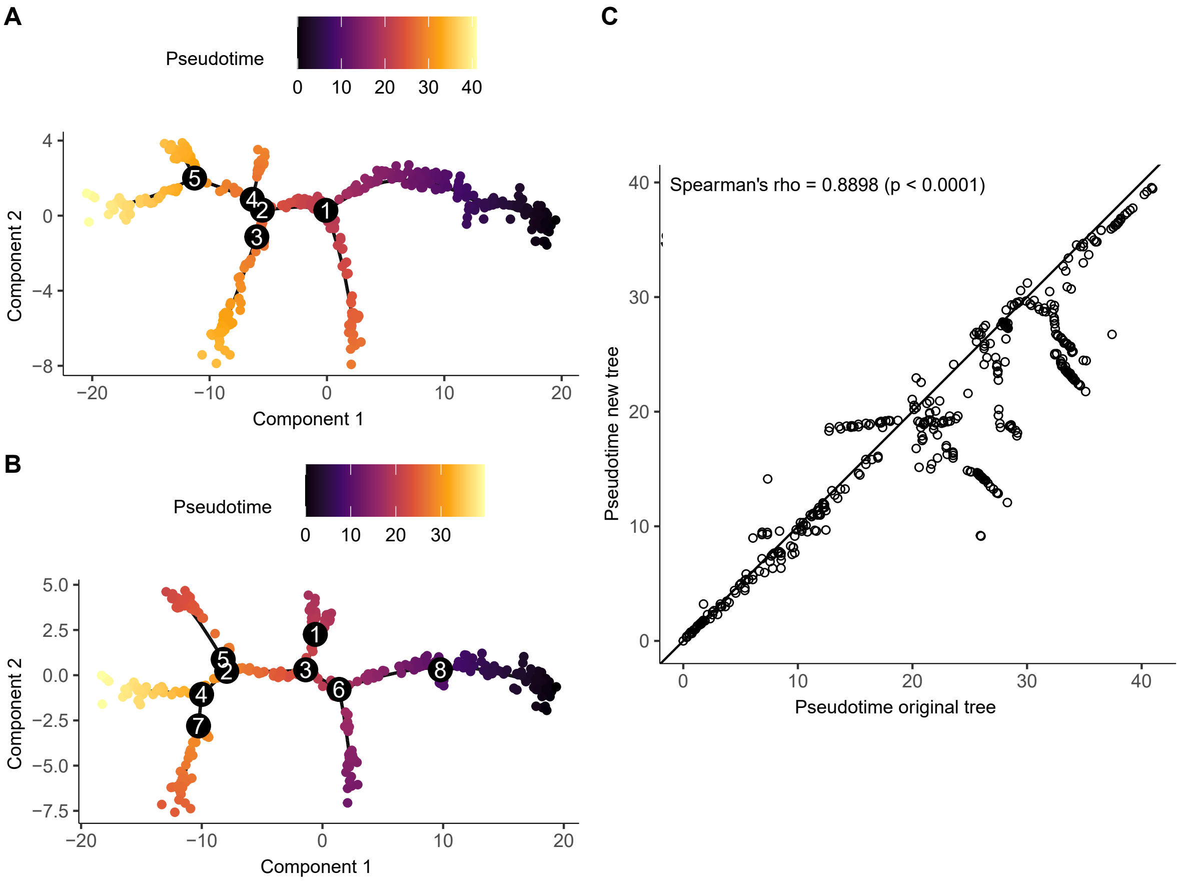

Supplementary Figure X. Relative positions of subjects in the original and unrelated subjects tree. The pseudotime calculated from the whole cohort tree (A) and that without unrelated subjects (B) shows that the subjects relative positions are highly correlated (Spearman's rho = 0.89) (C). This suggests that the tree generated after removing the related subjects was consistent with the one from the whole cohort.

Supplementary Table VII. Participant characteristics and CMR-derived cardiac measurements in UK Biobank. BSA, body surface area; concentricity, (left ventricular mass / left ventricular end-diastolic volume); CMR, cardiac magnetic resonance imaging; DBP, diastolic blood pressure; EDV, end-diastolic volume; EF, ejection fraction; ESV, end-systolic volume; FD, fractal dimension; LV, left ventricular; LVM, left ventricular mass; LVMI, left ventricular mass index (LVM/BMI); peak diastolic strain rate, PDSR; RA, right atrial; RV, right ventricular; SBP, systolic blood pressure; WT, wall thickness. *Medication for cholesterol, blood pressure, diabetes.

Characteristic	UKBB n=16,691
Female	8,775 (52.5)
Age at scan, y	55 ± 7.5
White	14,683 (87.9)
BSA, m^2	1.9 ± 0.2
LVEDV, ml	148 ± 33.5
LVESV, ml	60.4 ± 19
LVEF, ml	59.6 ± 6
LVM, g	86 ± 22.1
LVMI, g/m^2	45.8 ± 8.5
LV maximum WT, mm	9.4 ± 1.6
Mean apical FD	1.21 ± 0.05
Mean basal FD	1.19 ± 0.03
Mean global FD	1.17 ± 0.03
LV global radial strain, %	45 ± 8.3
LV global circumferential strain, %	-22.3 ± 3.4
LV global longitudinal strain, %	-18.5 ± 2.8
LV radial PDSR	-5.7 ± 2
LV longitudinal PDSR	1.7 ± 0.6
LV concentricity, g/mL	0.58 ± 0.08
Heart rate, min	69.5 ± 11.6
Hypertension	4,857 (29)
On medication*	2,241 (13.4)
SBP, mmHg	137.5 ± 18.1
DBP, mmHg	78.6 ± 9.9

Supplementary Table VIII. Characteristics of Singaporean HCM cohort. BSA, body surface area; SBP, systolic blood pressure.

Characteristic	Singaporean HCM n=60
Female	11 (11.7)
Age at scan, y	58.9 ± 20
Chinese	52 (86.7)
BSA, m^2	1.8 ± 0.2
SBP, mm Hg	137 ± 24.8
Genotype SARC-NEG	28 (46.7)
Genotype SARC-VUS	16 (26.7)
Genotype SARC-P/LP	16 (26.7)

Supplementary Table IX. Cumulative hazard model. All cause mortality in individuals with hypertrophic cardiomyopathy carrying pathogenic or likely pathogenic sarcomeric variants (SARC-P/LP) compared to those without variants in genes that may cause or mimic HCM (SARC-NEG) and those with variants of uncertain significance (SARC-VUS), adjusted for Age, Sex and Race. n = 436; $P = 0.002$. [1] HR = Hazard Ratio, CI = Confidence Interval

	Full model			Genotype only		
**Characteristic**	**HR[1]**	**95% CI[1]**	**p-value**	**HR[1]**	**95% CI[1]**	**p-value**
**P/LP**						
N	—	—		—	—	
Y	2.63	1.43, 4.86	0.002	2.62	1.42, 4.84	0.002
**Race**						
White	—	—				
Other	0.68	0.34, 1.37	0.3			
**Sex**						
F	—	—				
M	1.15	0.72, 1.85	0.6			

Supplementary Table X. Cumulative hazard model excluding SARC-VUS. All cause mortality in individuals with hypertrophic cardiomyopathy carrying pathogenic or likely pathogenic sarcomeric variants (SARC-P/LP) compared to those without variants in genes that may cause or mimic HCM (SARC-NEG), adjusted for Age, Sex and Race. N = 395; P = 0.003. [1] HR = Hazard Ratio, CI = Confidence Interval

	Full model			Genotype only		
**Characteristic**	**HR**[1]	**95% CI**[1]	**p-value**	**HR**[1]	**95% CI**[1]	**p-value**
**P/LP**						
N	—	—		—	—	
Y	2.66	1.43, 4.95	0.002	2.64	1.40, 4.98	0.003
**Race**						
White	—	—				
Other	0.68	0.30, 1.54	0.4			
**Sex**						
F	—	—				
M	1.26	0.77, 2.06	0.4			

Dimensionality reduction and unsupervised clustering of clinical features

Participant features comprised demographic data, clinical characteristics, CMR and echocardiographic measurements, and reported interventions and medicines (Supplementary Table XI). Missing values were inferred with the `mice` package for R.[63] Numerical features were converted to categorical variables by clustering groups of values into bins with a K-means algorithm.[64] All categorical variables were then transformed into binary variables with one-hot encoding.

Dimensionality reduction was performed on this collection of binary variables with UMAP (uniform manifold approximation and projection)[65] using the following parameters: Dice metric, 25 components, 8 neighbouring sample points and a minimum distance between points of 10^{-6}. Finally, unsupervised clustering was applied to the 25 resulting UMAP components with a K-means algorithm assessed and optimised with a silhouette score, revealing three clusters. Genotype status was found to be significantly associated with the clusters, using a χ^2 test. A post-hoc exact Fisher test was then performed to find cluster-specific enrichment. Adjustment for multiple testing was done with the Benjamini-Hochberg procedure, $P < 0.05$. Cluster 1 was significantly enriched with genotype-negative (NEG) subjects while cluster 3 was associated with genotype-positive (P/LP) and genotype-indeterminate (VUS) individuals. Feature importance from the initial set of participant data was assessed by applying a Kruskal-Wallis test for numerical features and a χ^2 test for categorical features. Significant associations (adjusted with the Benjamini-Hochberg method, $P < 0.05$) were further tested for cluster-specific enrichment: a Dunn test was used to test each pair of numerical features (Supplementary Fig. XIVA), while an exact Fisher test looked for one-vs-rest differences in categorical features (Supplementary Fig. XIVB).

The clustering revealed features characterising each group: 1) older female participants with lower body surface area (BSA), lower left ventricular (LV) volume, higher ejection fraction, hypertension, low activity score and on beta blockers and diuretic medications; 2) male participants with higher BSA, higher LV mass, higher LV maximum wall thickness, hypertension, moderate activity score and on medications for blood pressure (ACE/ARBs) and protective vascular (ASA/Clopi) medications; 3) younger participants with a family history of hypertrophic cardiomyopathy (HCM), no clinical cardiovascular risk factor and a high activity score.

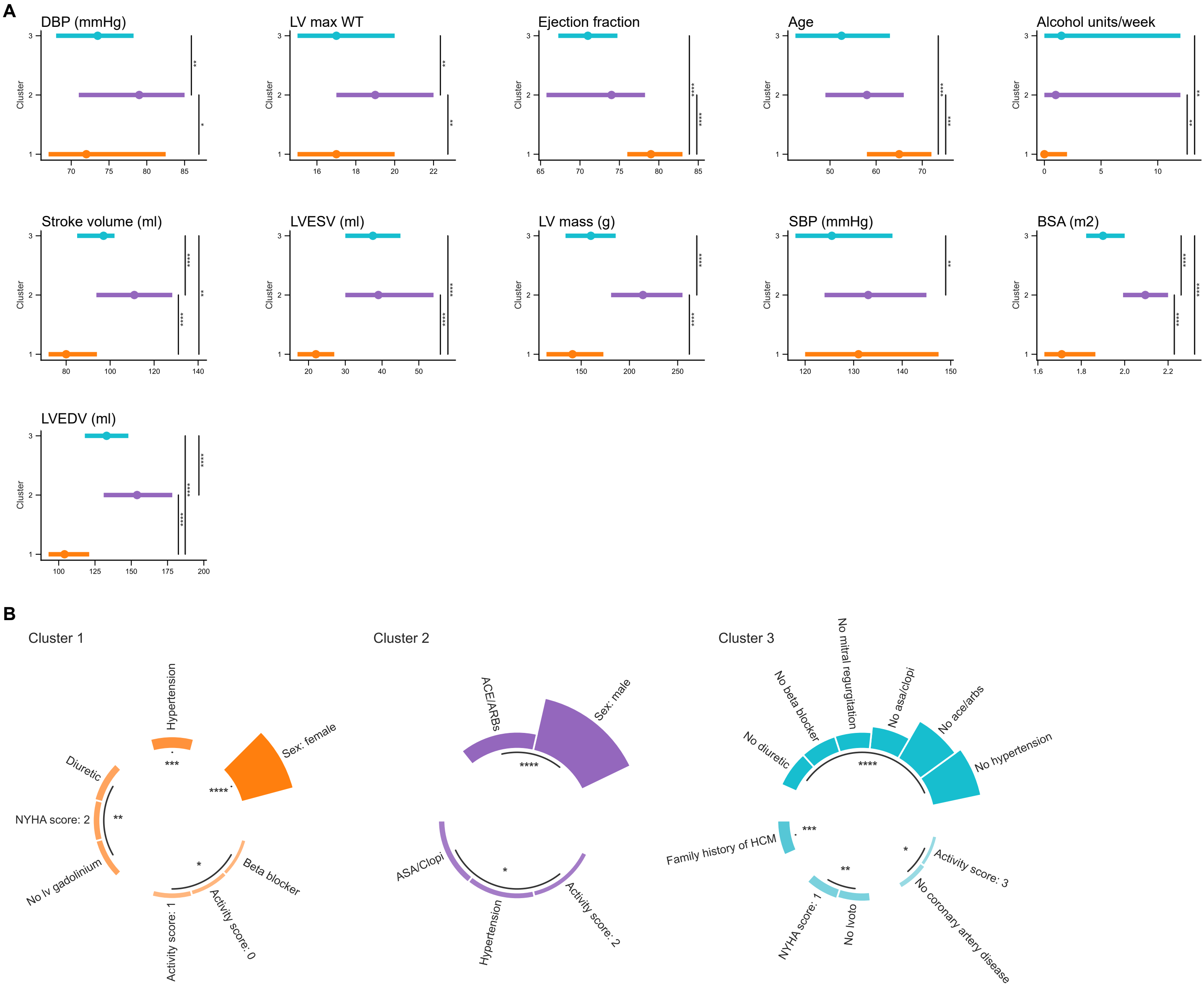

Supplementary Figure XI. Unsupervised clustering of clinical features and feature importance. a. Significant pairs of associations between identified clusters and numerical features from the initial set of data. The line represents the interquartile range and median value. **b.** Significant one-vs-rest associations between clusters and categorical features from the initial set of data grouped by significance level. The height of the curved bars illustrates the significance level ($-log_{10}P$). Only the significant pairs are reported with the symbols: $* \ P \leq 0.05; ** \ P \leq 0.01; *** \ P \leq 0.001; **** \ P \leq 0.0001, n = 436$.

Supplementary Table XI. Participant clinical features. MRI, magnetic resonance imaging; HCM, hypertrophic cardiomyopathy; SCD, sudden cardiac death CCS, Canadian Cardiovascular Society[66]; NYHA, New York Heart Association[67]; LV, left ventricular; RV, right ventricular; ACE, angiotensin-converting enzyme; ARB, angiotensin receptor blockers; ASA, acetylsalicylic acid.

**Demographics**	**Mean ± SD or n (%)**
Age at time of MRI scan (years)	57.3 (± 14.4)
Sex, male	310 (71.1%)
Ethnicity	
non-Finnish European	353 (81%)
South Asian	52 (11.9%)
African	16 (3.7 %)
Others	14 (3.2 %)
East Asian	1 (0.2 %)
**Clinical characteristics**	
Body surface area (m^2)	2 (± 0.3)
Diastolic blood pressure (mmHg)	76.2 (± 11.4)
Systolic blood pressure (mmHg)	133 (± 18.5)
Pulse rate (bpm)	70.1 (± 13.7)
Smoker	172 (39.4%)
Alcohol intake (units per week)	6.8 (± 12.3)
Activity score	
0	64 (14.7%)
1	68 (15.6%)
2	252 (57.8%)
3	49 (11.2%)
4	3 (0.7%)
Hypertension	175 (40.1%)
Diabetes mellitus	47 (10.8%)
Coronary artery disease	46 (10.6%)
Myocardial infarction	21 (4.8%)
Family history of HCM	85 (19.5%)
Family history of SCD	70 (16.1%)
CCS Angina Grading Scale	
0	57 (13.1%)
I	277 (63.5%)
II	88 (20.2%)
III	13 (3.0%)
IV	1 (0.2%)
NYHA Classification of Heart Failure	
No heart failure	48 (11.0%)
I	177 (40.6%)
II	176 (40.4%)
III	31 (7.1%)
IV	4 (0.9%)

**Measurements derived from MRI and echocardiogram**	**Mean ± SD or n (%)**
LV end-diastolic volume (mL)	137.6 (± 34.5)
LV end-systolic volume (mL)	37.3 (± 18.3)
LV stroke volume (mL)	100 (± 24.4)
LV ejection fraction	73.5 (± 8.5)
LV mass (mL)	189 (± 67)
LV maximum wall thickness (mm)	18.8 (± 4.5)
LV most affected segment	
Anterior	43 (12.0%)
Inferior	8 (2.2%)
Lateral	11 (3.1%)
Septal	295 (82.6%)
LV most affected level	
Base	210 (59.3%)
Mid	78 (22.0%)
Apex	66 (18.6%)
Mitral regurgitation	
None	203 (54.9%)
Minimal	124 (33.5%)
Moderate	40 (10.8%)
Severe	3 (0.8%)
LV gadolinium	
None	53 (14.0%)
Minimal	124 (32.7%)
Moderate	133 (35.1%)
Severe	69 (18.2%)
RV hypertrophy	45 (10.3%)
Coincident infarction	19 (4.4%)
LV outflow tract obstruction	120 (27.5%)
LV outflow peak velocity (m/s)	2.3 (± 0.7)
**Interventions and medicines**	
ACE inhibitors and ARBs	145 (33.3%)
ASA/clopidrogel	184 (42.2%)
Beta blocker	210 (48.2%)
Diuretic	66 (15.1%)
